# Adaptive Normal Mode Sampling (aMDeNM) Enhances Exploration of Protein Conformational Space and Reveals the Functional Role of Frequency Coupling

**DOI:** 10.1021/acs.jctc.6c00398

**Published:** 2026-06-11

**Authors:** Pedro T. Resende-Lara, Maurício G. S. Costa, Balint Dudas, Janka Czigleczki, Erika Balog, David Perahia

**Affiliations:** † Laboratoire de Biologie et Pharmacologie Appliquée, 4919 Ecole Normale Supérieure Paris-Saclay, Gif-sur-Yvette 91190, France; ‡ Programa de Computação Científica, Vice-Presidência de Educação, Informação e Comunicação, Fundação Oswaldo Cruz, Av.Brasil 4365, Rio de Janeiro 21040-900, Brazil; § Department of Biophysics and Radiation Biology, 37637 Semmelweis University, Tűzoltó u. 37-47, Budapest 1094, Hungary

## Abstract

Proteins exhibit a diverse range of structures and dynamics that are critical to their biological function. These dynamic processes span a broad spectrum of time scales and are influenced by environmental factors, including temperature, solvent composition, and the presence of binding partners or membranes. Efficient exploration of protein conformational space is essential for understanding their functional mechanisms, but this remains challenging because of the high dimensionality of the energy landscape. Our group has previously developed the molecular dynamics with excited normal modes (MDeNM) method, which is based on the kinetic excitation of normal modes (NMs) during molecular dynamics simulations. Here, we developed an adaptive extension of the method (aMDeNM), where the motions described by preselected directions of low-frequency NMs are dynamically adjusted throughout the simulation. By coupling low-frequency NM excitation with adaptive directional adjustments, aMDeNM facilitates extensive exploration of the energy landscape, overcoming the constraints of fixed, rectilinear displacements and alleviating structural stresses and environmental resistance. The method was tested on three structurally diverse test systems: T4 lysozyme, human calmodulin, and*Staphylococcus aureus* monofunctional transglycosylase. Our results demonstrate improved conformational sampling compared with standard MD and other enhanced sampling methods. Additionally, spectral analysis of structural oscillations along the pathways using fast Fourier transform revealed the role of low-frequency vibrations in critical conformational changes and highlighted the influence of the surrounding environment on protein dynamics. This work provides a robust framework for studying large-scale protein motions and their functional implications within complex biological environments. Importantly, aMDeNM requires only an initial structure without the need to specify predefined target states, distinguishing it from many biased sampling techniques that rely on predefined target conformations. The aMDeNM code and usage instructions are available at https://github.com/pedro-tulio/amdenm.

## Graphical abstract

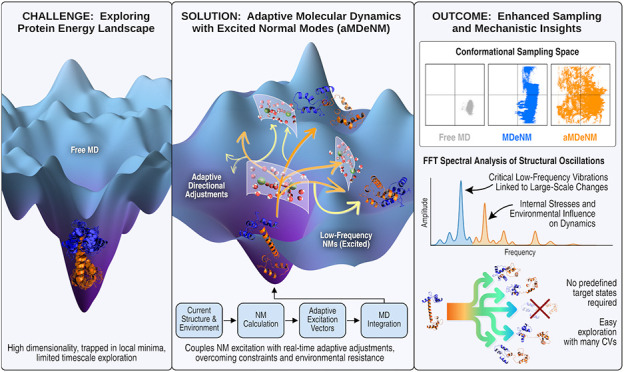

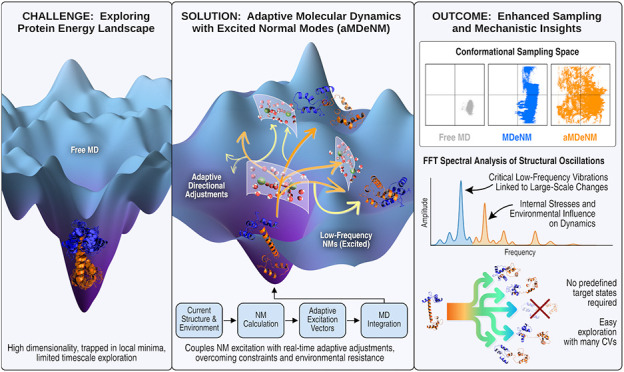

## 1 Introduction

The diversity of protein structures and dynamics dictates their wide range of functions in biological systems.^1–6^ The functional internal dynamics of proteins occur on time scales ranging from femtoseconds to milliseconds, and beyond, encompassing a broad spectrum of motions from local fluctuations to large-scale collective movements.^7,8^ The free energy landscape (FEL) and associated pathways, which can be derived from these conformational distributions, are crucial for understanding processes such as protein folding, ligand binding, and macromolecular interactions, ultimately shaping protein function.^9,10^ FEL analysis broadens the traditional structure–function paradigm by linking thermodynamics and kinetics,^11–13^ yet their application remains challenging due to the high dimensionality of the FEL. These analyses depend heavily on developing methods to improve or accelerate conformational sampling in biomolecular simulations.

Over the past few years, several techniques have emerged to overcome the sampling problem, leading to a myriad of philosophies about how to tackle the issue. A straightforward approach to undertake this problem is developing specialized hardware to compute larger simulations at faster rates. This was done by Shaw et al. in 2008, when the Anton supercomputer was presented.^14^ Although this specialized machine is more than 10 times faster than general-purpose supercomputers,^14–16^ its architecture and rigid algorithmic constraints restrict its adaptability to novel force field parametrizations.^17–19^ Finally, Anton remains commercially prohibitive and institutionally inaccessible for the broader research community due to its proprietary ASIC implementation.^20^

In addition to the hardware approach, most methods use software solutions to address the sampling problem. A widely used class of these methods aims to enhance conformational sampling by either increasing the simulation temperature or modifying the potential energy functions.^21–26^ However, these methods demand extensive validation and may still not be sufficient for a comprehensive exploration of the FEL. Although accelerating barrier crossing, these methods do not eliminate the intrinsic high dimensionality of free energy landscapes, hidden slow degrees of freedom, parameter sensitivity, or force-field limitations, so important basins and pathways may remain unsampled or artificially distorted.^27,28^ Moreover, because these approaches alter kinetics and often require reweighting to recover unbiased thermodynamics, they demand extensive validation to ensure convergence and physical reliability yet may still fail to fully reconstruct the true free energy landscape.^29^

Another class of enhanced sampling methods improves conformational exploration by utilizing collective variables (CVs) to guide the sampling process,^30–34^ driving the system along predefined directions. Consequently, the accuracy of these methods depends on the choice of CVs and whether they adequately represent the key slow degrees of motions capturing the underlying biological processes. Deep learning techniques have shown promise for optimizing CVs,^35,36^ though they require extensive data. CVs are generally obtained by diagonalizing large matrices, such as force constant matrices in Normal Mode Analysis (NMA) or positional covariance matrices from Molecular Dynamics (MD) simulations in Principal Components Analysis (PCA). For large systems, computing the low-frequency normal modes (NMs) using a physical force field (PFF) while accounting for all atomic degrees of freedom is computationally demanding. Despite their slow convergence rates, iterative methods are well-suited for such large-scale calculations.^37^ To reduce computational cost, it is common to consider subsets of atoms, either by grouping rigid blocks of atoms^38^ or by selecting only representative atoms, such as C-alpha atoms in proteins, within an elastic network model (ENM) to calculate the modes. Each of these methods has its own inherent limitations. For instance, one major drawback of PCA is that the principal movement directions depend highly on the simulation length. Therefore, capturing all relevant motions may require long simulations to ensure comprehensive sampling. Furthermore, PCA only captures linear motions.

On the other hand, NMA (PFF or ENM) takes only a single structure as input to describe low-frequency directions, which align remarkably well with experimentally observed large-scale conformational changes.^39^ Nonetheless, extending NM movements over large amplitudes can introduce significant anharmonic couplings, which may limit the accuracy of the approach. This is because Cartesian NM vectors describe only rectilinear motions, overlooking extensive domain rotations or structural deformations. Although NMA using dihedral angles is an alternative method, it is more difficult to apply to macromolecules and their complexes due to the need to consider all atoms.^40–42^ Thus, large displacements along collective directions require either energy minimization or MD simulation, reducing the effectiveness of conformational sampling. In Cartesian space, another approach is to update the NM vectors throughout the sampling process to capture significant nonlinear deformations.^43,44^ However, this approach requires repeated matrix diagonalization, which can be computationally expensive for large systems. Additionally, it may introduce structural instability due to abrupt changes in NM vectors when following a given motion direction. Iterative and adaptive approaches have gained increasing interest recently for their ability to overcome these limitations.^45,46^

Here, we introduce an adaptive approach that extends and greatly improves the accuracy of the method we developed earlier, Molecular Dynamics with Excited Normal Modes (MDeNM),^47^ which couples local and global motions by kinetically exciting NMs (or a combination) within MD simulations to propagate conformational changes. Other NM-based methods follow a similar philosophy of exploring conformational landscapes within a collective space.^44,48,49^ Their particular characteristics and performance have been reviewed elsewhere.^50^ In our adaptive approach (aMDeNM), the direction of excitation is dynamically adjusted during an MD simulation without imposing any bias on the potential energy. This adaptive strategy helps alleviate structural stresses observed when a fixed excitation direction is used straightforwardly, as well as environmental constraints, allowing for a more extensive exploration of the FEL.

We tested our method on three diverse molecular systems: bacteriophage T4 lysozyme (T4L), human calmodulin (CaM), and *S. aureus* monofunctional transglycosylase (MTG). Our results show great improvement in conformational sampling compared with simulations without directional corrections. Additionally, we performed spectral analysis of the adaptive motions to gain insights into the contributions of modes at the low-frequency end of the vibrational spectrum, revealing their involvement during the critical conformational changes. This analysis used the fast Fourier transform (FFT) of the displacement fluctuations recorded along the adaptive paths during the simulations. The resulting spectra revealed how the kinetic energy introduced along the path is absorbed and redistributed across other degrees of freedom within the protein. Furthermore, we examined the extent to which the surrounding environment influences the spectral distribution, providing a deeper understanding of its role in protein dynamics.

The following sections begin with a brief overview of the MDeNM method, in which kinetic energy is injected periodically, thereby allowing the system to relax between the excitations. Then, we introduce aMDeNM, the approach that incorporates adaptive motions. Our improved method maintains a quasi-constant excitation energy while continuously adjusting the excitation direction throughout the simulation. For comparison, we additionally explored an alternative approach, cMDeNM, which maintains a quasi-constant excitation energy along a fixed excitation direction. We then applied these methods to selected proteins and analyzed the results.

## 2 Theory

### 2.1 Molecular Dynamics with Excited Normal Modes without and with Adaptive Motions

In MDeNM, additional velocities are assigned to atoms based on the components of a selected NM vector (or a combination of NM vectors) in an MD simulation. This kinetic excitation directs the system along collective motions, facilitating the propagation of conformational changes. While these approaches have been used to explore the low-frequency subspace, higher-frequency motions associated with localized and internal movements across different regions may also play a critical role in conformational exploration. However, because the excitation’s kinetic energy rapidly dissipates into the medium during the MD simulation, multiple excitations are required to sustain the displacement. Between two excitations, corresponding to a relaxation period, the system releases excess energy until the next excitation occurs. This sequence of excitations and relaxations enables quasi-adiabatic motion, preventing excessive accumulation of kinetic energy that could be detrimental. Indeed, the excitation energy must be relatively low to avoid significant internal structural distortions, unfolding, and exploration of high-energy regions of the FEL.

The new aMDeNM approach dynamically adjusts both the direction of motion and the kinetic excitation energy rather than relying on fixed directions and bursts of high kinetic energy. By maintaining a nearly constant, low excitation kinetic energy over short periods, during which the direction of motion is continuously adjusted, the system can access previously unexplored regions of the FEL. These small adaptive adjustments in collective movement directions enable a more extensive exploration of conformational space, leading to structural changes that would not have been possible along a fixed direction defined by the initial NM vectors, and enhancing the system’s flexibility and responsiveness to emerging stresses. The quasi-constant low excitation energy also provides a sustained driving force along newly adapted motion directions, further promoting efficient conformational sampling.

### 2.2 aMDeNM Flowchart

The aMDeNM approach is illustrated in Figure 1 and is described in detail in the following sections.

**Figure 1:**
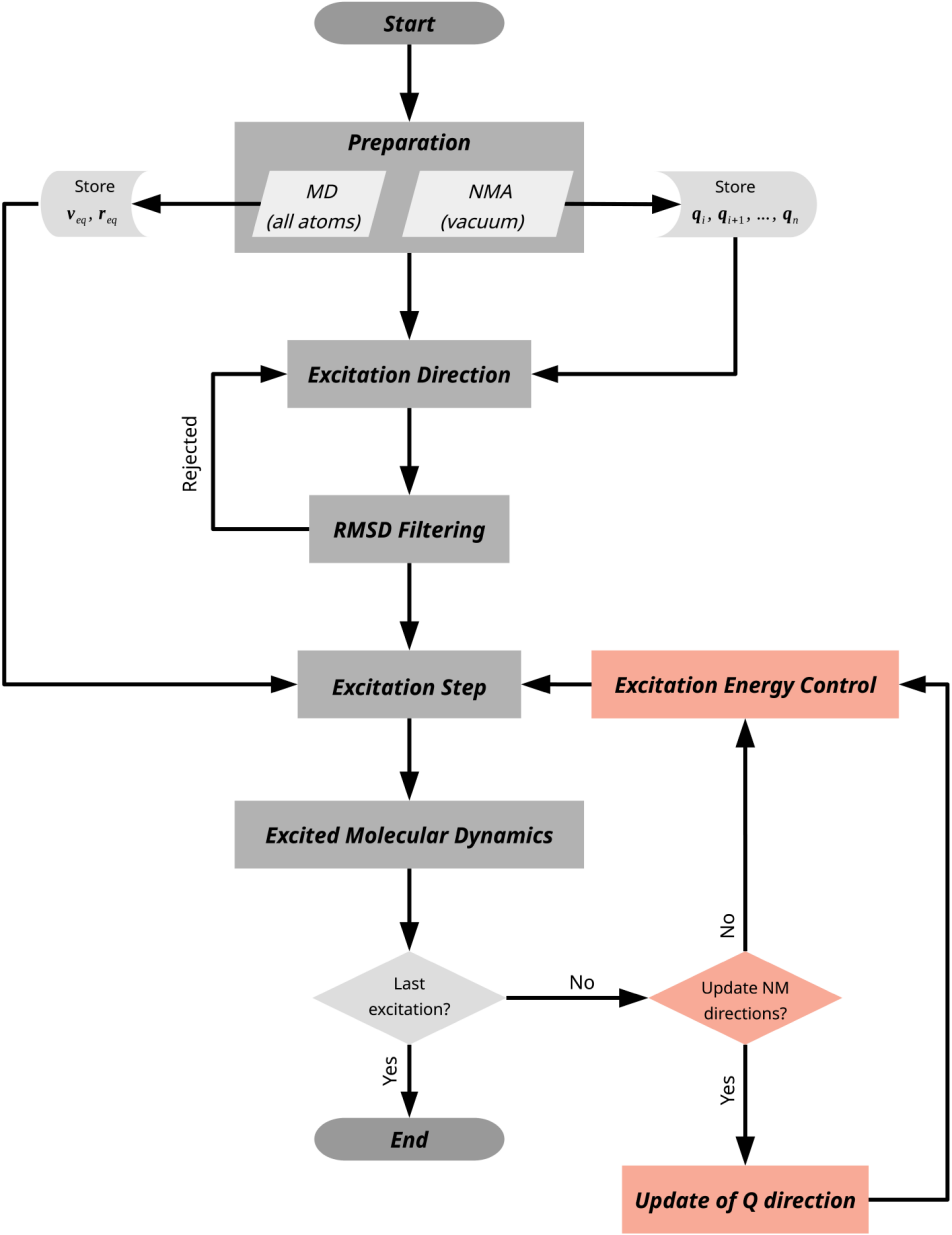
aMDeNM flowchart. The preparation step consists of running a short MD and computing the normal modes of the minimized structure, resulting in the initial positions (**r** *_eq_*) and velocities (**v** *_eq_*), as well as the directions for further exciting the system (**q** *_1···n_*). Next, the excitation vector (**Q**) is generated as a random linear combination of different NMs. In a multireplica procedure, each replica has a different **Q** vector. These vectors are then compared to one another to retain only those that are sufficiently different, thereby avoiding unnecessary computation (RMSD filtering step). Once a vector is retained, it is multiplied by a scalar (λ) that determines the additional energy (*ΔE*) set by the user, resulting in the excitation velocities (**v** *_ex_*), which are ultimately added to the current ones (**v** *_eq_*). The simulation is then started. After each excitation, the energy along the vector **Q** is evaluated and, if necessary, rescaled to match the user-specified value. Finally, the system’s effective displacement vector is compared to the excitation vector used. If the angular deviation between them reaches or exceeds 60°, the excitation vector is updated accordingly for the next excited simulation (see the text for more details).

#### 2.2.1 System Preparation

This step is a prerequisite for performing the MDeNM simulations. It involves independently (i) performing a short MD equilibration run to store the final atomic velocities **v**_eq_ and positions **r**_eq_ and (ii) computing the normal modes from the final energy-minimized MD coordinates, retaining vectors from the low-frequency end of the vibrational spectrum.

#### 2.2.2 Determination of the Initial Excitation Vectors

An excitation vector **Q** is defined by the normalized linear combination of *n* normal mode vectors **q**_i_, given by

1 $$Q=\frac{1}{\sqrt{\overset{n}{\underset{i=1}{\sum }}}\alpha_{i}^{2}}\overset{n}{\underset{i=1}{\sum }}\alpha_{i}q_{i}$$

where α_*i*_ represents a uniformly distributed random number between – 0.5 and 0.5. Then, a set of randomly distributed excitation vectors is generated, and a smaller subset is selected to ensure a proper coverage of the multidimensional space (see RMSD filtering below).

#### 2.2.3 RMSD Filtering

We previously developed an RMSD filtering procedure to optimize the selection of excitation directions for MDeNM simulations.^47^ Briefly, this procedure consists of accepting or rejecting a given **Q**_i_ vector from the randomly generated ensemble based on a user-defined RMSD threshold. This is an iterative procedure that starts by performing 1 Å geometric displacements from the initial structure along each **Q**_i_ vector successively; a vector is only selected if the corresponding displaced structure’s RMSD with the previously chosen structures exceeds a predefined threshold.

The RMSD between two structures is given by the equation:

2 $$\mathrm{RMSD}=\sqrt{\frac{1}{N}\overset{N}{\underset{i=1}{\sum }}}{\left(r_{i}^{\prime }-r_{i}\right)}^{2}$$

where *N* is the number of Cα atoms, and ***r*** ^*’*^_*i*_ and ***r***_*i*_ are the positions of the *i* ^*th*^ atom in the two structures being compared.

#### 2.2.4 Initial Excitation

The excitation energy (*E*_*exc*_) governs the speed and extent of exploration within the conformational space and must be sufficiently high to promote significant collective motion. Given a user-defined *E*_exc_ value and a **Q** excitation vector, the excitation velocity vector (**V**_exc_) is computed as

$$V_{exc}=\sqrt{2}E_{exc}M^{-\frac{1}{2}}Q$$ 3

At the start of the simulation, the Cartesian atomic velocities related to the excitation (**V**_exc_) are added to the current velocities of the equilibrated structure (**V**_curr_):

4 $$V_{tot}=V_{curr}+V_{exc}$$

Subsequently, the velocities are adjusted during successive excitations to maintain the same excitation energy level.

#### 2.2.5 Adjusting the Kinetic Energy along the Excitation Vector

As given by eq 5, the kinetic energy along the normalized **Q** direction is calculated by projecting first the current velocities to the excitation direction **Q**:

5 $$V_{p}=\left(V_{\mathrm{curr}}Q\right)Q$$

where **V**_p_ and **V**_curr_ are the *3N-*dimensional vectors of the projected and current atomic velocities, respectively.

The kinetic energy along the excitation direction is thus given by eq 6

6 $$E_{k}=\frac{1}{2}V_{p}^{T}MV_{p}$$

where **M** is the diagonal mass matrix.

At the beginning of each short simulation interval, the remaining excitation energy (*E*_*k*_) is adjusted to the desired excitation level (*E*_*exc*_) by modifying the atomic velocities. The adjustment is made as given in eq 7:

7 $$V_{\mathrm{new}}=V_{\mathrm{curr}}+\left(V_{\mathrm{exc}}-V_{p}\right)$$

The importance of controlling kinetic energy injection is further detailed in the Supporting Information and illustrated in Supporting Figure 1.

#### 2.2.6 Determining the Direction of the Successive Excitation Vectors

The changes in the direction of the excitation vectors for the subsequent *n* ^*th*^ excitations (**Q**_**n+1**_) depend on specific parameter values obtained along the trajectory followed in the **Q**_**n**_ direction. The first excitation vector, **Q**_**1**_, corresponds to a given combination of normal modes of the starting structure. Figure 2 illustrates the excitation vector correction during aMDeNM simulations. At the end of each excitation step, the system is aligned to the initial conformation in order to control rotation and translation. Then, the direction of the subsequent excitation vector is defined based on two parameters: the first relates to the effective displacement $l_{n}$ along **Q**_n_ during the *n* ^*th*^ excited dynamics. $l_{n}$ is obtained by projecting the effective mass-weighted root-mean-square displacement (MRMS) vector **d**_n_ onto the normalized mass-weighted excitation vector **Q**_n_. eq 8 defines how **d**_n_ is determined:

8 $$d_{n}=M^{1/2}\left({\langle r\rangle }_{n}-r_{n}^{0}\right)$$

where the brackets ⟨ ***r*** ⟩*_n_* specify the average position of the structures over the last 0.1 ps obtained, and $r_{n}^{0}$ is the starting position in the *n* ^*th*^ excitation, respectively.

**Figure 2:**
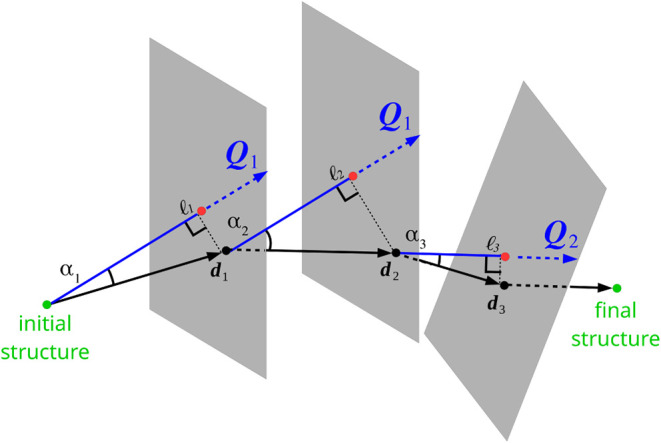
Adaptive correction of the excitation vector during aMDeNM simulations. The vectors **Q** *_n_* represent the excitation directions, while **d** *_n_* denotes the effective mean displacement during the corresponding excitation MD run. Once a given MRMS displacement threshold is reached for $l_{n}$ along **Q** *_n_*, the effective displacement vector **d** *_n_* is examined to determine whether to define a new excitation vector or maintain the current one. The update of the **Q** vector is governed by the angular deviation α*_n_* and the displacement along **Q** as specified in eq 10. The gray planes, perpendicular to their respective excitation vectors, indicate the end of short simulation runs. In the example shown, the first evaluation results in *cos α*_1_ *≥ 0.5*, meaning the excitation direction remains the same as the previous excitation **Q**_1_. In the next evaluation, however, *cos α*_2_ *< 0.5,* prompting the definition of a new excitation vector **Q**_2_ having the same direction as **d**_2_. This adaptive approach enables the exploration of new directions while accounting for the internal stresses and constraints imposed by the medium on protein motions.

The second parameter relates to the relative deviation of the vector **d**_n_ with respect to vector **Q**_n_, evaluated by the angle α_*n*_ between them. More precisely, we consider the *cos α*_*n*_ obtained by taking the scalar product of these vectors after normalizing **d**_n_, as given by eq 9 (note that **Q**_n_ is already a unit vector):

9 $$\cos\alpha_{n}=\frac{d_{n}Q_{n}}{∥d_{n}∥}$$

To decide whether the direction of the excitation vector will be modified in the subsequent excitation, we apply the following rule: the direction is updated only if the displacement $l_{n}$ , along **Q**_n_, is larger than a threshold value $l_{n}$ , and cos α is lower than a threshold value *cos α*_*c*_. The conditions for choosing the excitation vector between **d**_n_ and **Q**_n_ for the next simulation are defined by eq 10:

$$Q_{n+1}=\{\begin{array}{ccc} Q_{n}, & \mathrm{if}\;l\leq l_{c}\\ Q_{n}, & \mathrm{if}\;l>l_{c} & \wedge \cos\alpha \geq \cos\alpha_{c}\\ d_{n}, & \mathrm{if}\;l>l_{c} & \wedge \cos\alpha <\cos\alpha_{c} \end{array}$$ 10

Neutron scattering studies and MD-based analyses consistently show that large-amplitude, low-frequency protein motions are substantially anharmonic, and the harmonic approximation is generally considered valid only for motions below 1 Å in amplitude.^51–53^ The threshold values $l_{c}$ and *cos α*_*c*_ were determined through a series of simulations of T4 lysozyme, in which both constants were systematically varied and evaluated. We first set a range of values for $l_{c}$ and observed that *cos α*_*c*_ varies linearly with $l_{c}$ . Taking the average *cos α*_*c*_ value and changing the l threshold resulted in simulations that either changed the excitation direction too early (giving the system no time to explore it) or too late (structurally deforming the protein). However, an optimal combination was retained from these simulations, 0.5 *m* ^1/2^Å (*m* being the atomic mass unit), for $l_{c}$ and 0.5 for *cos α*_*c*_. The detailed procedure is discussed in the Supporting Information and depicted in Supplementary Figure 2. The relationship between l and *cos α*_*c*_ is also analyzed.

The threshold $l_{c}$ is introduced to account for displacements exceeding thermal fluctuations. Otherwise, the simulation loses precision in determining the new directions. Likewise, it is also necessary that the change of orientation be sufficiently large to proceed with the update. If the change of orientation is large, but the displacement is small, or vice versa, the update is not performed due to excessive uncertainty caused by fluctuations. This linear behavior between $l_{c}$ and *cos α* indicates that the system has demonstrably entered the anharmonic regime, where the original NM eigenvector combination ceases to be a valid descriptor of the local energy landscape. Therefore, the first condition ensures the system has actually moved into a meaningfully different region of conformational space (i.e., small fluctuations around the reference are not mislabeled as anharmonic drift). The second condition ensures the direction of that motion has genuinely diverged from the excitation vector. Together, they implement a two-dimensional criterion in space (displacement and deviation), preventing both premature corrections (small motion) and neglecting drift (large angular deviation without sufficient displacement).

## 3 Results

In the following, we will compare the conformational sampling achieved by aMDeNM with that of MDeNM (as published by Costa et al.),^47^ cMDeNM (controlled energy injection without direction update), and free molecular dynamics on the three systems T4L, CaM, and MTG. Different reasons led us to this choice: 1) T4L exhibits large-scale dynamics primarily described by only two low-frequency normal modes, making it an ideal test case for adjusting and validating the simulation parameters of aMDeNM, as well as for straightforwardly describing its dynamics; 2) CaM possesses many low-frequency normal modes that contribute to a complex domain-closing movement, making the protein particularly well suited for aMDeNM; moreover, even enhanced sampling simulations have hardly been successful in capturing domain-closing motions;^44,54,55^ and 3) MTG is a transmembrane protein, meaning that its motions can be influenced by external stresses from the heterogeneous environment (membrane and water). This makes it an excellent candidate for testing aMDeNM under more complex conditions. The choice of relevant internal coordinates for each system is detailed in the Supporting Information and illustrated by Supplementary Figure 3. As input, we used 48 isotropic combinations of modes 7 and 8 (the two lowest frequency normal modes) to generate excitation vectors for each system. In this section, we also rationalized the differences between the three MDeNM approaches for T4L in light of the vibrational spectrograms obtained.

### 3.1 aMDeNM Notably Extends the Conformational Space Explored by T4L

The hinge bending and domain twisting motions account for approximately 90% of T4L’s overall dynamics.^56^ After 125 ns of free MD from an open conformation, the sampling remained limited to a minimum energy well between states most of the time, mainly neighboring the closed state conformation (Figure 3a). Despite the relatively long simulation time, not all experimental structures were covered by free MD. In contrast, WTMetaD significantly improved sampling, exhibiting good sampling in both open and closed states (Figure 3b). MDeNM also enabled a broader exploration of conformational space in comparison to free MD, with open and closed states well sampled within 12 ns (48 replicas of 250 ps each), as shown by the high-density regions in the map (Figure 3c). This aligns with previous studies showing MDeNM’s ability to efficiently shift between protein macrostates.^47,57,58^ Importantly, the most frequently visited conformations corresponded to the lowest potential energy states relative to the minimized structure (Figure 3d). With cMDeNM, we observed even greater sampling (Figure 3e), but the increased motion led to structural distortions, including a slight loss of secondary structure (Supplementary Figure 4). Moreover, the closed conformation was less populated, and the potential energy of sampled states was higher than in MDeNM (Figure 3f). This suggests that cMDeNM retains more energy, reducing relaxation and enabling transitions across larger energy barriers, though not necessarily into biologically relevant states. On the other hand, aMDeNM densely populated either the open or closed macrostates (Figure 3g). Dynamic adjustment of excitation directions minimized structural deformation, preserving the secondary structure (Supplementary Figure 4). As in MDeNM, lower-energy states were concentrated along the transition path between open and closed conformations (Figure 3h). Notably, aMDeNM also captured wide-open and twisted structures with low relative energies (the upper-right corner of Figure 3g), further enhancing conformational diversity. Table 1 presents the minimal RMSD between our simulation conformations and experimentally open (178L_A) and closed (152L_F) states. Although presenting good overall sampling, WTMetaD achieved the worst RMSD results. On the other hand, MDeNM approaches presented best results for open (aMDeNM) and closed (MDeNM) states.

**Figure 3:**
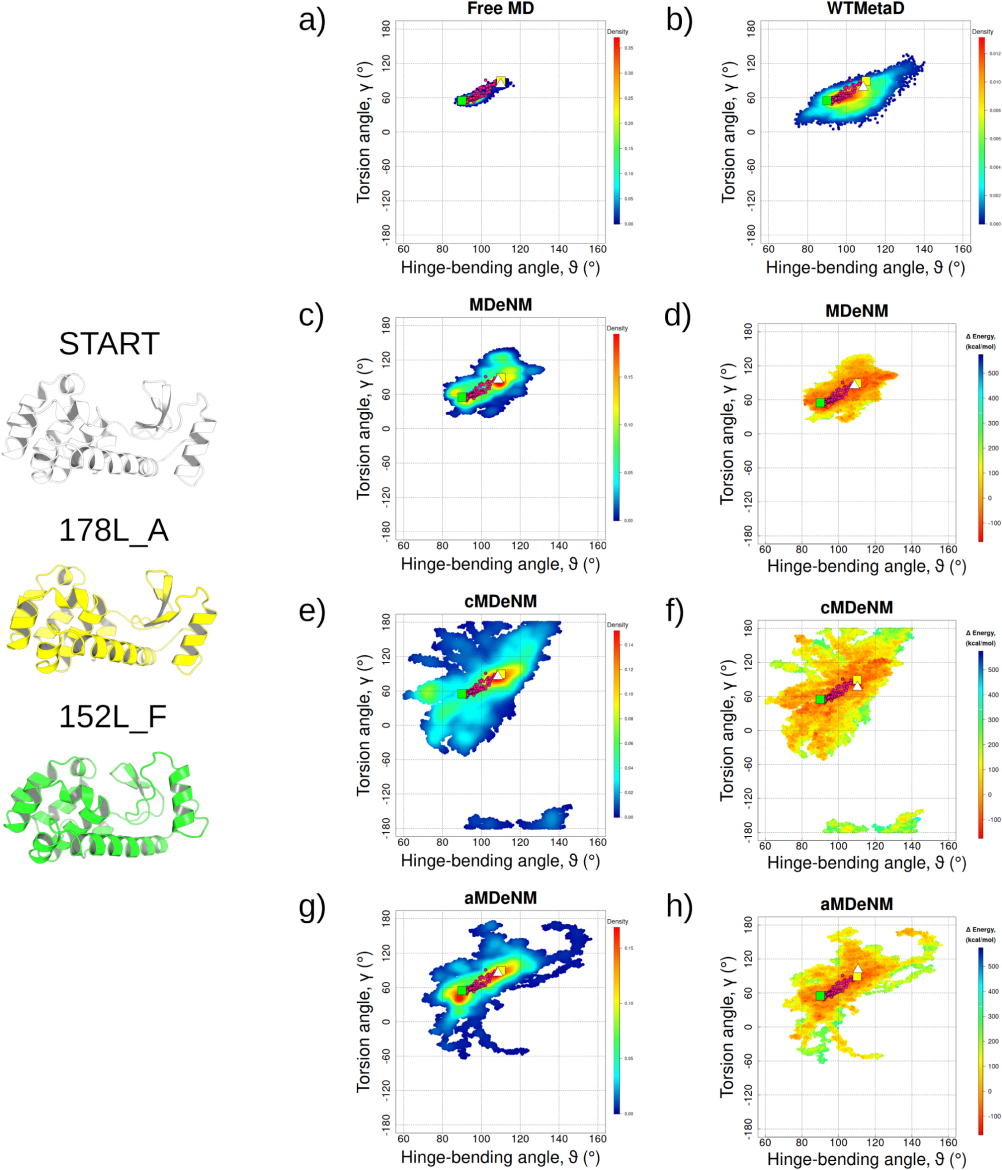
T4L Conformational sampling of T4L. Density scatter plots illustrating the conformational sampling of T4L in different simulations: **a)** free MD, **b)** WTMetaD, **c)** MDeNM, **e)** cMDeNM, and **g)** aMDeNM. Less frequently sampled regions are shown in blue, while the most visited areas are in red. Potential energy differences (E_initial_ *–*E_current_) are shown for **d)** MDeNM, **f)** cMDeNM, and **h)** aMDeNM. Magenta circles represent experimental structures. The white triangle indicates the simulation’s initial structure. Yellow and green squares indicate open and closed structures, respectively, as illustrated in the bottom-left panels.

**Table 1:** Minimal RMSD to Selected T4L Experimental Structures (in Å)

Structure	Free MD	WTMetaD	MDeNM	cMDeNM	aMDeNM
**152L_A**	0.821	0.881	**0.818**	0.862	0.827
**178L_A**	0.857	0.925	0.728	0.705	**0.656**

### 3.2 Low-Frequency Excitation Promotes Higher Frequencies’ Activation with aMDeNM

Because of the successive updates to the excitation direction, the final excitation vector can differ significantly from the initial one. This results in the activation of different internal motions throughout the excited trajectory, as depicted in Figure 4. The first three panels of Figure 4 show simulations where only normal mode 7 (hinge bending motion) was used to excite the system. In the MDeNM approach, T4L transitioned from the open to the closed state, encountered a barrier, and oscillated around the same conformation without further progression (Figure 4a). In contrast, the cMDeNM approach crossed this barrier, continued along the closing motion, and sampled a broader conformational space (Figure 4B). However, this approach also introduced some structural distortions at the end point.

**Figure 4:**
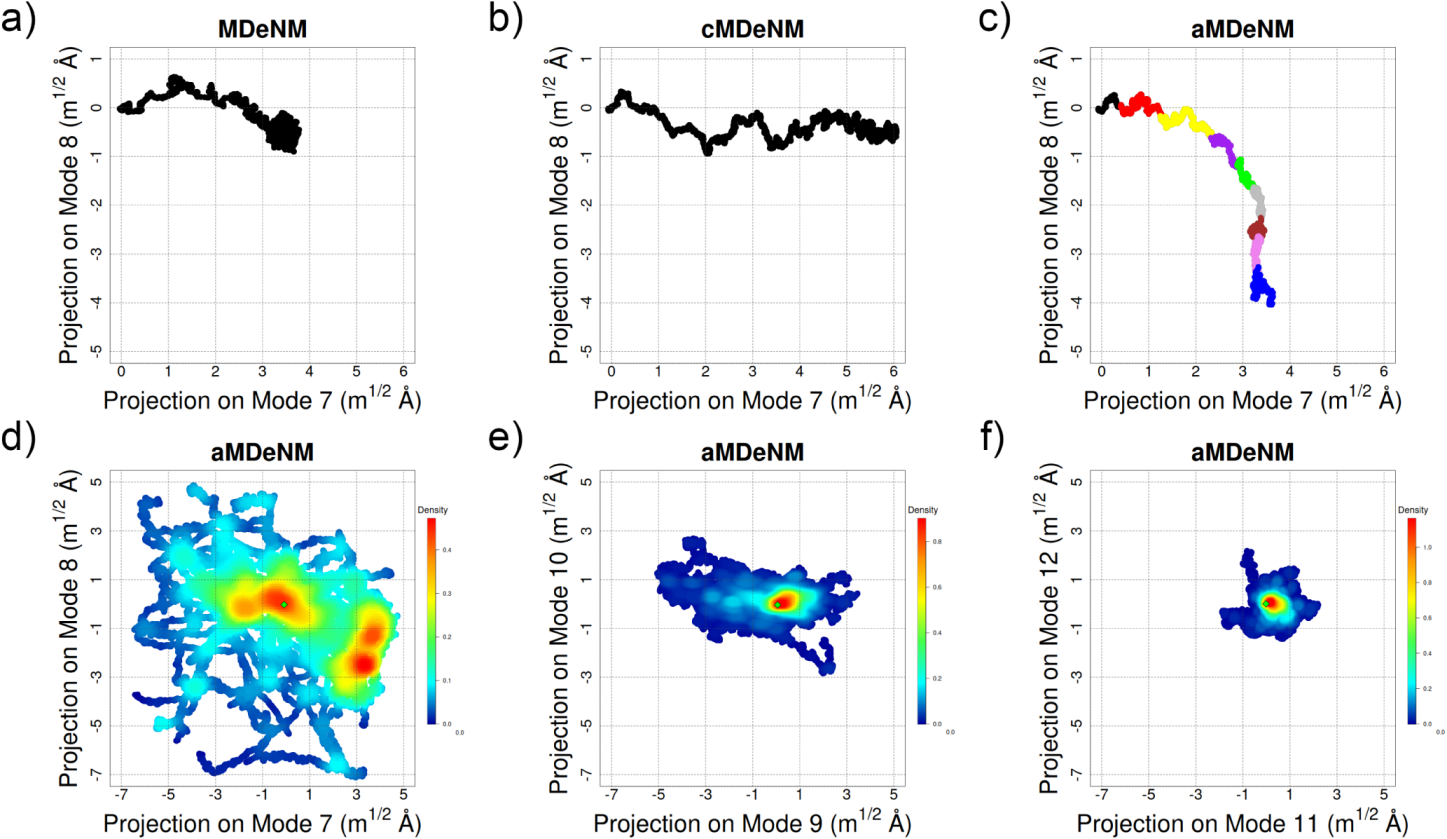
Projection of MDeNM, cMDeNM, and aMDeNM trajectories of T4L onto the normal modes. In the top three maps, excitation was performed along normal mode 7, and the trajectories projected onto the surface defined by normal modes 7 and 8: with **a)** MDeNM, **b)** cMDeNM, and **c)** aMDeNM. For the latter, each color represents a different excitation vector adaptively generated during the simulation. In the bottom three maps, the simulations were performed with aMDeNM using 48 isotropic combinations of modes 7 and 8, and the projections are displayed as population densities onto the surface defined by **d**) modes 7 and 8, **e)** modes 9 and 10, and **f)** modes 11 and 12. The green diamonds represent the MD starting conformation.

Interestingly, mode 8 became fully activated during the adaptive approach with aMDeNM (Figure 4c). As the trajectory progressed, the influence of mode 7 gradually diminished, giving way to mode 8. Notably, despite this shift, the final displacement along mode 7 remained almost the same as in the original approach, MDeNM. However, the conformational pathway taken was significantly different. In addition, aMDeNM reached the closed conformation slightly more effectively than the original approach, with a Cα-RMSD of 1.15 Å compared to 1.21 Å relative to the closed structure. In contrast, neither MDeNM nor cMDeNM exhibited significant contributions from mode 8 (torsion) during the trajectory, with observed slight changes likely attributable to thermal oscillations.

Considering all 48 combinations of modes 7 and 8, we identified two distinct, densely populated regions visited during the simulations (Figure 4d). Additionally, we observed significant contributions from modes 9 and 10 (Figure 4e) and modes 11 and 12 (Figure 4f), despite none of these modes being explicitly excited at the beginning. This suggests that the protein naturally activates multiple internal motions, described by other modes, to facilitate relaxation along the trajectories.

### 3.3 Spectral Analysis of T4L

We projected the trajectories onto their respective excitation vectors to analyze the spectral characteristics of the conformational pathways in each of the MDeNM methods. We then computed the spectra of these projected fluctuations over short time intervals throughout the simulation using the FFT. For cMDeNM and MDeNM, projections were performed onto the single initial excitation vector, whereas for aMDeNM, they were computed along the successive excitation vectors. The resulting spectra are presented in Figure 5.

**Figure 5:**
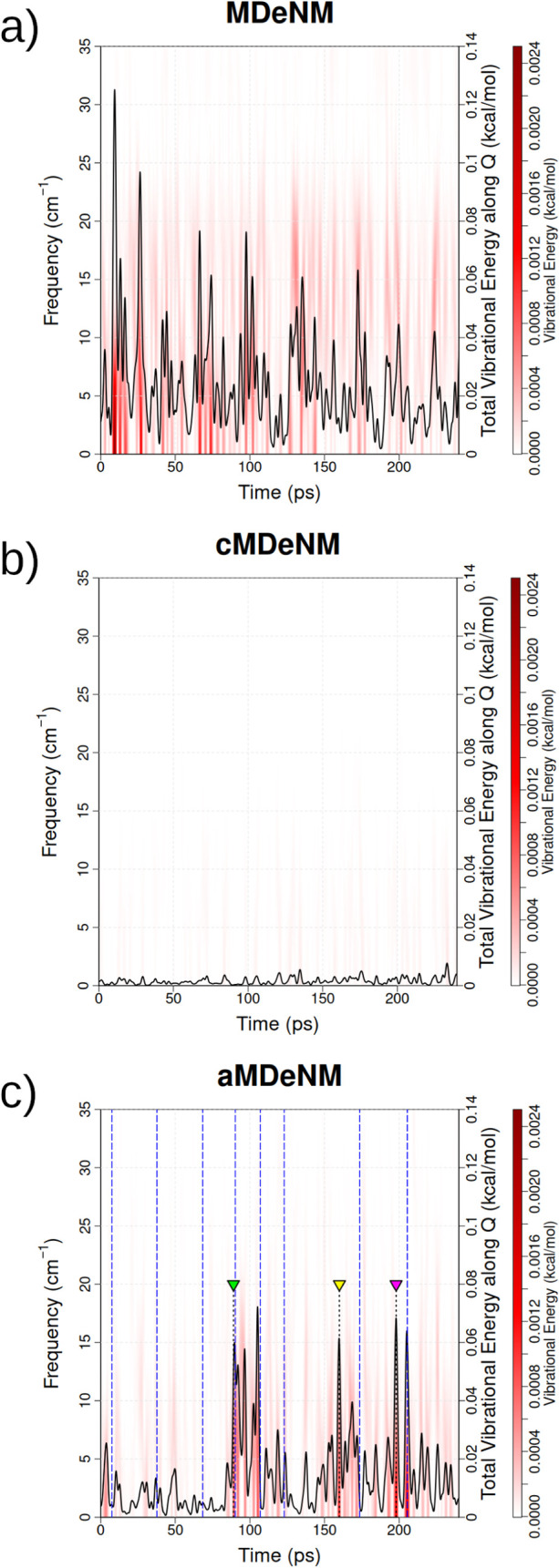
Spectral analysis of MDeNM, cMDeNM, and aMDeNM trajectories of T4L along normal mode 7. Spectrograms of trajectory projection fluctuations along mode 7 for **a)** MDeNM, **b)** cMDeNM, and **c)** aMDeNM. The vibrational energies associated with different frequencies are represented as heatmaps. The black curve shows the total vibrational energy up to 35 cm^–1^ over time. Blue vertical lines indicate the moments when the excitation direction is updated. Significant conformational changes are shown as green, yellow, and magenta triangles.

#### 3.3.1 Spectral Energy Distribution Across Methods

In MDeNM simulations (Figure 5a) we observed that vibrational energy is broadly distributed across a wide range of low frequencies. This finding aligns with previous studies, indicating rapid dissipation of excitation energy during the relaxation phase.^47^ In contrast, cMDeNM showed minimal energy dispersion across low frequencies (Figure 5b). This is likely due to cMDeNM’s controlled energy injection, which maintains a nearly constant low excitation energy with a minimal relaxation time, preventing significant energy transfer to other vibrational modes.

Interestingly, aMDeNM exhibited an intermediate energy distribution between those of MDeNM and cMDeNM (Figure 5c). Its spectrum reveals phases of intense energy dispersion only in some periods in the trajectory. For example, energy dispersion remained low during the 10 to 85 ps interval, followed by bursts of high dispersion between 85 and 110 ps (marked by intense red regions) around 90, 160, and 200 ps. The strong dispersion phases closely coincide with major conformational transitions such as domain shifts or torsions: (i) the first one occurs when the closing motion stops as the protein can no longer follow this direction without undergoing steric clashes between the N- and C-terminal domains (green triangle, Figure 5c); (ii) the second corresponds to the N-terminal domain torsion (yellow triangle, Figure 5c); and (iii) the third is when the two domains start to drift apart from each other (magenta triangle, Figure 5c). Although aMDeNM maintains a constant energy update like that of cMDeNM, it also redistributes kinetic energy across different vibrational modes by adjusting the excitation direction. This redistribution is particularly enhanced when the system encounters internal stresses. As shown in Figure 4e–f, the redistribution of vibrational energy can be interpreted as mode–mode coupling across a broad frequency range, reinforcing the hypothesis that major conformational changes require the participation of multiple vibrational modes.

It is worth noting that in MDeNM, intense vibrational energy bands appear more frequently than in aMDeNM, likely due to multiple barrier crossings as the system is unable to adjust its direction of motion to avoid them. Moreover, the intensity of these bands gradually decreases as the system evolves, reflecting its difficulty in undergoing conformational changes. In contrast, this was not observed in aMDeNM, where high-intensity bands appear toward the end of the trajectory, indicating new conformational transitions such as the one at 200 ps, which involves a domain shift.

#### 3.3.2 Total Vibrational Energy Along Reaction Pathways

Beyond energy distribution, we also analyzed the total vibrational energy along the reaction pathways across the considered frequency range (black curves in Figure 5). MDeNM simulations exhibited higher overall energy values than cMDeNM and aMDeNM. This is because MDeNM continuously injected greater kinetic energy at each excitation cycle, whereas in cMDeNM and aMDeNM, energy levels were adjusted to lower values through successive updates. The relaxation time between energy injections in MDeNM was also insufficient for complete energy dissipation. It is worth noticing that aMDeNM displayed higher kinetic energy values than cMDeNM. This is closely related to its dynamic adaptation of excitation vectors, which enhances couplings with vibrational modes that absorb substantial energy, thereby requiring larger energy injections during updates. Consequently, the most pronounced energy peaks in aMDeNM coincide with significant directional changes.

### 3.4 Greatly Improved Description of CaM Motions by aMDeNM

We investigated the conformational sampling of CaM using 48 combinations of the two lowest-frequency normal modes (NM7 and NM8). This analysis focused on the two structural descriptors given in Supporting Information and Supplementary Figure 3. The initial structure exhibited an extended conformation, with the linker fully structured as an α-helix. During 125 ns of free MD, CaM primarily fluctuated around this extended structure, failing to capture the entire experimental conformational landscape, as shown in Figure 6a. While MDeNM significantly improved the sampling, it did not fully reproduce all the experimentally observed conformations (Figure 6c).

**Figure 6:**
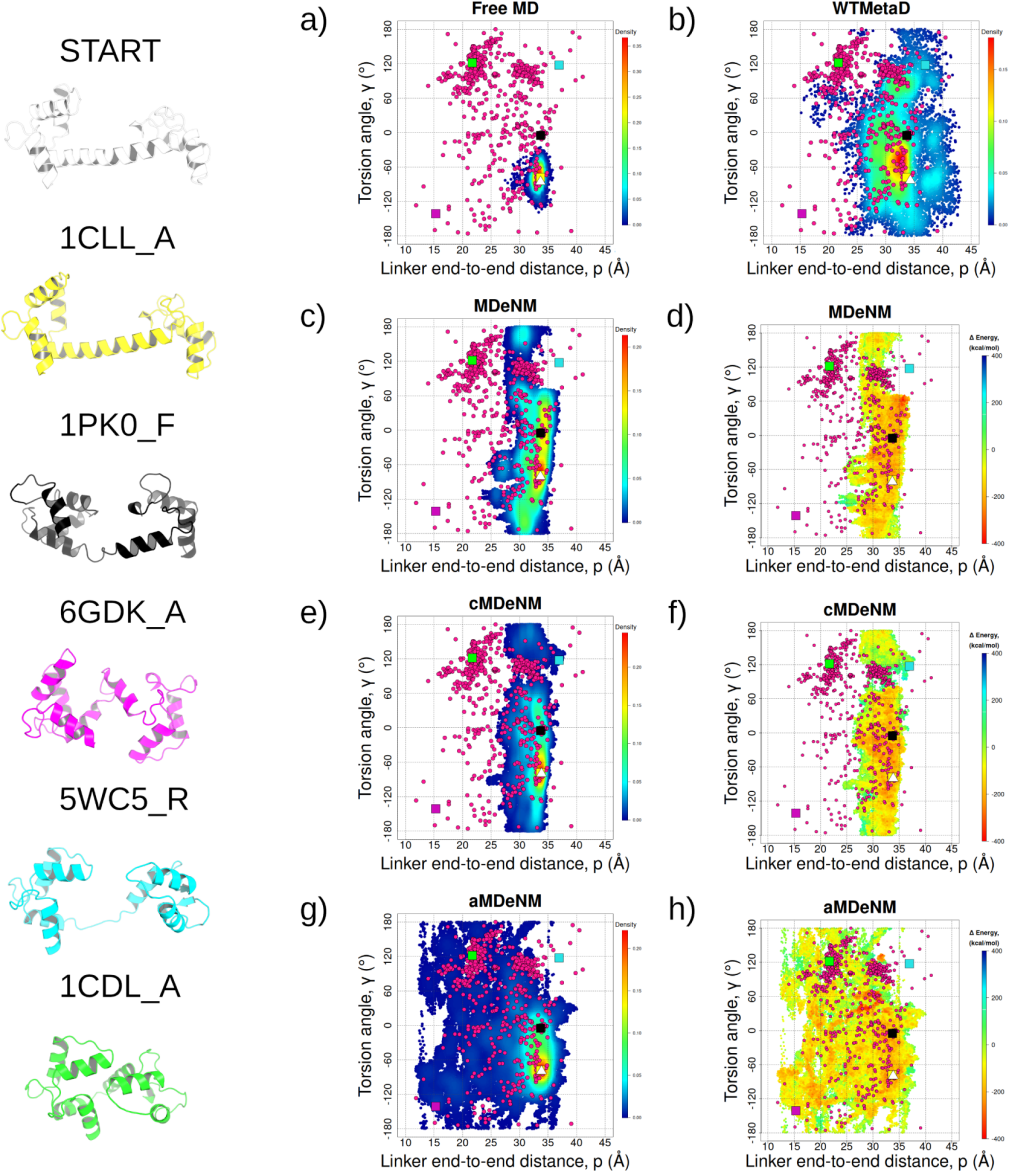
aMDeNM significantly enhances CaM sampling. Density scatter-plots illustrating the conformational sampling of CaM using **a)** free MD, **b)** WTMetaD, **c)** MDeNM, **e)** cMDeNM, and **g)** aMDeNM. The least-sampled regions are shown in blue, while the most frequently visited regions appear in red. Potential energy differences of the protein (E_initial_ *–*E_current_) calculated in vacuum are displayed for **d)** MDeNM, **f)** cMDeNM, and **h)** aMDeNM. Magenta circles represent experimental structures. The white triangle indicates the initial structure of the simulations. Colored squares highlight experimental structures with distinct conformations, as illustrated in the accompanying cartoon representations.

The cMDeNM approach exhibited a similar sampling pattern to MDeNM but enhanced exploration in a small, specific region near the structure 5WC5_R (the upper-right corner, Figure 6e). This structure corresponds to the Ca^2+^–CaM complexed with a small-conductance Ca^2+^–activated K^+^ channel. The linker is partially structured in this conformation, and the domains are twisted by approximately 120°.

In contrast, WTMetaD has greatly increased CaM sampling compared to the previous approaches (Figure 6b). Although this impressive enhancement, WTMetaD struggled to visit compact conformations, such as those similar to 6GDK_A and 1CDL_A (magenta and green squares in Figure 6, respectively). This was achieved by aMDeNM, which sampled nearly the entire experimental landscape (Figure 6g). Here, the adaptive approach successively changes the excitation direction, allowing the protein to overcome structural constraints that would otherwise be unattainable with a fixed excitation vector. This broader exploration is particularly notable in a highly flexible protein like CaM, demonstrating the method’s effectiveness in capturing complex, nonlinear motions. Interestingly, the region near structure 5WC5_R (upper right corner of Figure 6g) was not visited in the aMDeNM simulation. Since only the WTMetaD and cMDeNM simulations accessed this region, this suggests that energy barriers prevent sampling these conformations. WTMetaD simulations are designed to surpass the energy barriers by adaptively adding a repulsive bias potential along selected collective variables. The bias discourages revisiting low-free-energy regions, forcing the system to explore higher-energy configurations and thus cross barriers efficiently. Similarly, cMDeNM advances the structure along a more linear trajectory, with nearly constant kinetic excitation energy, a minimal relaxation time, and limited deviation compared to the other MDeNM approaches. These characteristics likely enabled it to overcome these barriers and reach the 5WC5_R region. Consistent with observations for T4L, the most densely sampled areas across all approaches corresponded to regions of the protein’s lowest potential energy when considered alone (Figure 6d, f, and h).

As previously discussed, the highly flexible CaM linker enables the protein to adopt a wide range of conformations. This flexibility is somewhat reflected in the broad distribution of the two structural descriptors, as shown in Figure 6. To better evaluate these complex motions, we analyzed the RMSD of the generated structures and compared them to representative experimental structures. This assessment provides a robust measure of the conformational sampling achieved by different MDeNM approaches.

Notably, aMDeNM significantly improved the overlap between experimentally observed and theoretically generated structures compared to MDeNM and cMDeNM as shown in Table 2. When comparing the probability density distributions obtained with each method, it is evident that aMDeNM surpasses the sampling provided by other approaches in practically all scenarios as shown in Figure 7 (only marginal differences compared to WTMetaD in 1CLL_A and 1PK0_F configurations). Moreover, secondary structure analysis indicates that aMDeNM-generated structures are more closely aligned with experimental data than those generated by the other approaches. However, some regions retain more residues in an α-helical conformation than observed experimentally (see Supporting Information).

**Figure 7:**
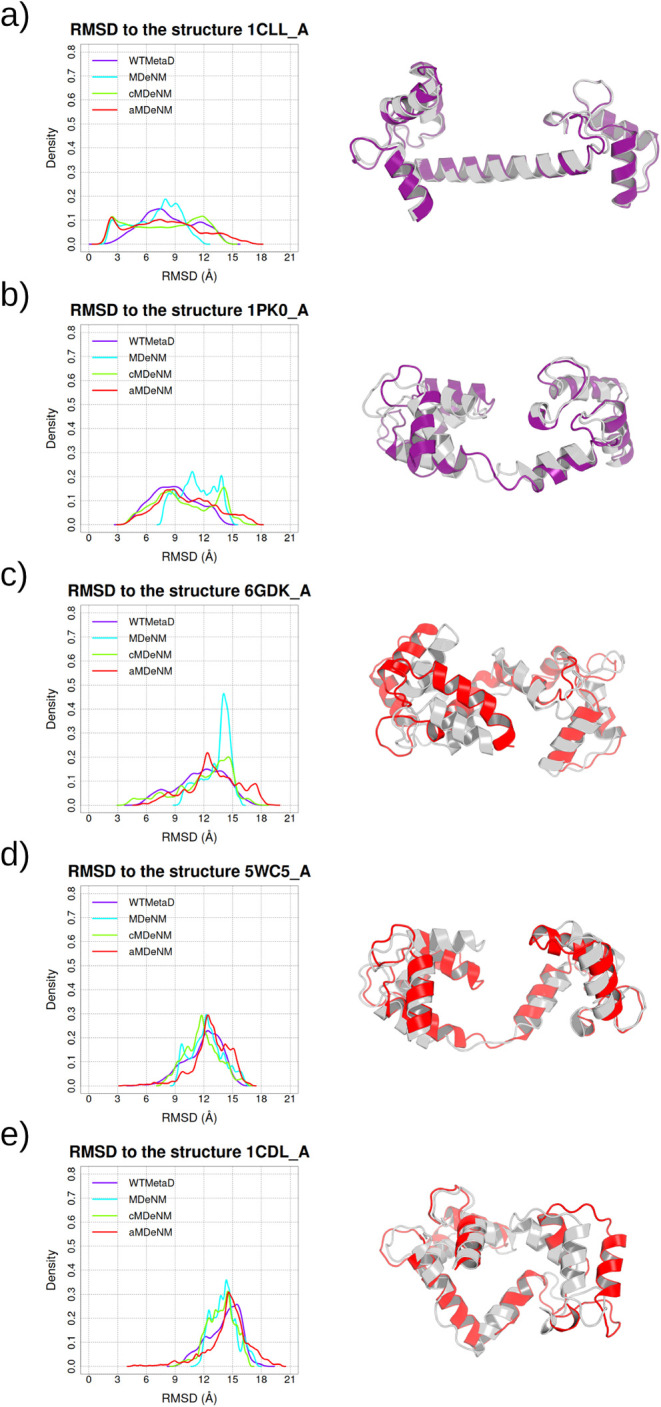
Probability density function (PDF) of RMSD distributions with respect to CaM representative experimental structures. RMSD PDF plots and their structural overlap between **a)** 1CLL_A, **b)** 1PK0_F, **c)** 6GDK_A, **d)** 5WC5_R, and **e)** 1CDL_A. Experimental structures are shown in gray, and their counterparts from the simulations presenting the lowest RMSD are colored according to the PDF curve. Both cMDeNM and aMDeNM displayed broader conformational sampling than free MD and MDeNM when evaluated against representative experimental structures.

**Table 2:** Minimal RMSD to Selected CaM Experimental Structures (in Å)

Structure	Free MD	WTMetaD	MDeNM	cMDeNM	aMDeNM
**1CLL_A**	1.748	**1.199**	1.739	1.547	1.321
**1PK0_F**	5.346	**3.616**	7.610	3.806	3.689
**6GDK_A**	10.347	5.159	9.770	8.009	**3.998**
**5WC5_R**	9.634	4.772	8.962	7.526	**3.586**
**1CDL_A**	12.052	8.905	10.994	8.844	**4.435**

### 3.5 MTG: TM Helix Orientation, Stability and Broader Sampling of Globular Domains

The transmembrane (TM) helix plays a crucial role in MTG’s enzymatic activity by ensuring proper membrane orientation.^59^ To evaluate the protein’s insertion into the membrane, we analyzed the bending and rotational behavior of the TM helix (see Supplementary Figure 3 for the descriptors). Across all simulations, the bending angle remained between 140° and 180° (Figure 8a–d). However, notable differences were observed in the rotational behavior. The TM helix exhibited limited rotation, approximately 20°, in free MD and MDeNM simulations (Figure 8a and b, respectively). In contrast, cMDeNM simulations showed a significantly broader rotational range, reaching 60° (Figure 8c). This behavior aligns with the characteristics of the NM vectors, which indicate high flexibility in the TM helix, as they were computed without the membrane embedding. The constant excitation energy of cMDeNM, lacking adaptive mechanisms to counteract membrane-induced stress, amplified these motions. In aMDeNM, adaptive changes in excitation directions stabilized the TM helix, reducing excessive fluctuations (Figure 8d). Meanwhile, the sampling of MTG’s globular domains was significantly enhanced in aMDeNM (Figure 8h) compared to cMDeNM (Figure 8g) and, most notably, to MDeNM (Figure 8f) and free MD (Figure 8e).

**Figure 8:**
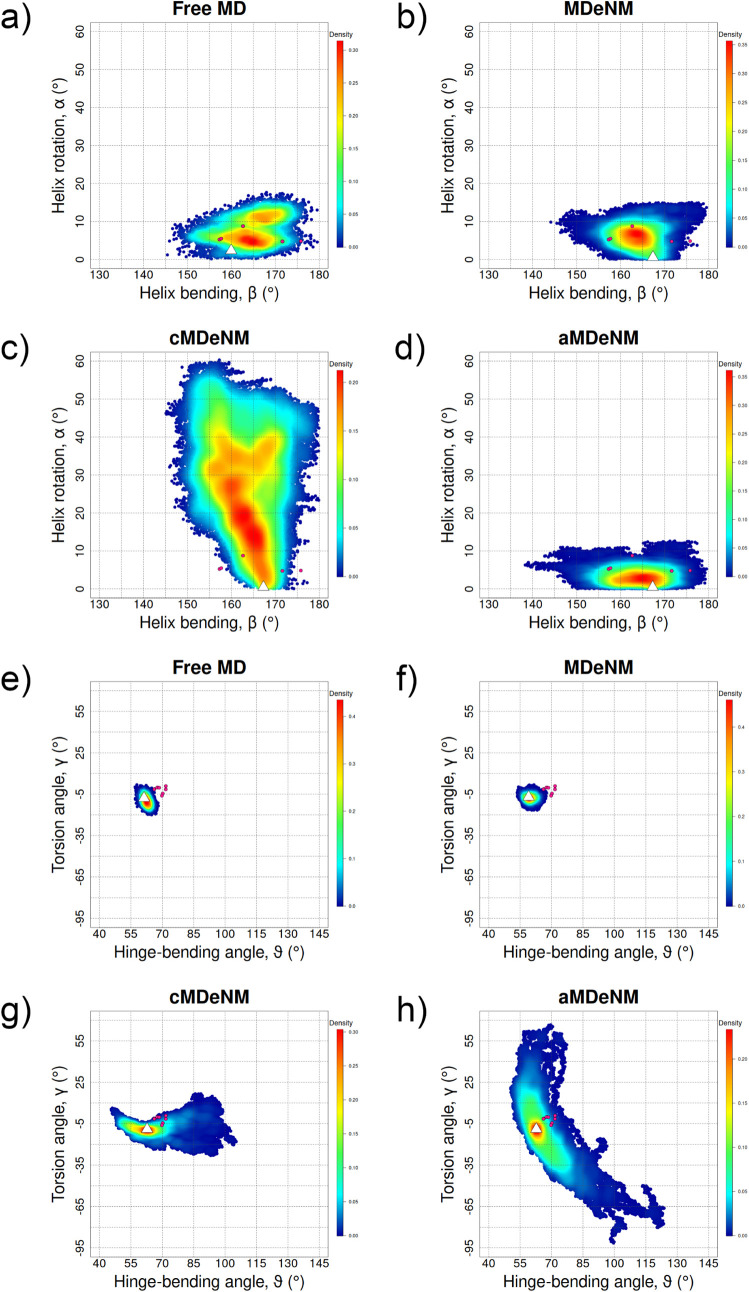
aMDeNM accounts for structural constraints imposed by the environment. The bending and rotation of the TM helix reflect its orientation within the membrane. **a)** Free MD and **b)** MDeNM presented a good sampling of the experimental structures. **c)** In contrast, cMDeNM exhibits excessive TM helix rotation, indicating exaggerated motions from the computed NMs. **d)** aMDeNM, however, maintains a sampling pattern similar to free MD and MDeNM, aligning well with experimental data. Regarding the globular domain of MTG, **e)** free MD and **f)** MDeNM showed limited conformational sampling, while **g)** cMDeNM expanded the explored conformations, though not as effectively as **h)** aMDeNM. Overall, these results demonstrate that aMDeNM incorporates structural and environmental constraints during conformational sampling.

## 4 Discussion

We introduced novel enhancements to the original MDeNM method, including self-regulated energy injection and adaptive excitation directions, while accounting for natural constraints imposed by the protein structure and environment. These improvements effectively prevented structural distortions during the simulation, enabling more accurate protein conformational sampling.

Our results showed that, similar to MDeNM, cMDeNM, and aMDeNM required significantly less computational time than both free MD and WTMetaD simulations while substantially enhancing the sampling of the conformational landscape. To be precise, only 12 ns of aMDeNM sampling yielded much better results than 250 ns of free MD and WTMetaD (Figures 3, 6, and 8). These findings align with prior studies, such as those by Zhang et al.,^33^ who have used ANM-accelerated MD (ACM) or PCA-driven approaches (ACM-PCA) to improve sampling.^60^ They have shown that recomputing ANM or PCA modes during simulations improved sampling for T4L while maintaining secondary structure integrity. More recently, a study demonstrated that a few ENM modes were very effective for exploring large protein motions and capturing transitions between macrostates.^61^ For T4L, only the three lowest-frequency modes were sufficient to describe transitions between the closed (PDB entry 177L) and open (PDB entry 178L) states. Our approach further showed that excitation of just two modes (7 and 8) was already sufficient due to the indirect activation of higher modes.

CaM exhibits intricate conformational dynamics essential for its regulatory functions across numerous cellular pathways. Several advanced sampling methods have been developed and applied to elucidate these dynamics. Among them, we can mention 1) NMA integrated tempering simulations (NMA–ITS),^62^ which integrates NMA with MD, using a biased potential to facilitate transitions between predefined macrostates. In contrast, aMDeNM enhanced sampling efficiency without relying on prior knowledge of macrostates or necessitating the recomputation of normal modes during each excitation cycle; 2) ClustENM, an iterative technique that utilizes anisotropic network model (ANM) modes to deform protein structures, generating conformational subspaces through successive steps. The resulting conformers are minimized using an implicit water model.^44^ ClustENMD uses a similar protocol but employs short molecular dynamics to relax the representative structures generated by the ANM deformation;^48^ 3) Similarly, Saldaño et al.^43^ have introduced a method employing NMA to produce realistic ensembles that depict protein equilibrium dynamics at specific temperatures, functioning in both biased and unbiased manners. They observed that a few mid-frequency modes were required to efficiently reproduce the CaM conformational landscape, leading them to complementary simulations that improved sampling coverage. This finding aligns with studies suggesting that a few global modes derived from elastic network models (ENMs) can effectively capture protein functional dynamics. Our findings are consistent with these results. However, we only needed two modes to obtain a better description of the CaM conformational landscape, whereas other methods require 3 to 30 modes.^43,44^ Notably, during aMDeNM simulations, the higher frequency modes were dynamically activated and effectively incorporated into the excitation direction. It demonstrated superior performance, particularly in capturing collapsed structures. This is especially interesting when considering the “curse of dimensionality”, in which the computational cost to explore the conformational space along *d*-dimensional CV space scales exponentially with *d*. WTMetaD is only effective when *d* is small^63^ and is rarely effective beyond *d*≈3–4 CVs. With aMDeNM, one can operate with high dimensionality through normal modes combination and adaptive activation of mid-frequency modes along the trajectory.

Their lipid membrane environment significantly influences the intricate dynamics of transmembrane proteins. Traditional computational methods, such as normal-mode analysis (NMA), often compute protein motions in a homogeneous dielectric medium, potentially overlooking membrane-specific constraints. This discrepancy has led to the development of methods integrating membrane effects into simulations, providing a more accurate depiction of protein behavior.^39,64^ In line with this, in aMDeNM simulations of MTG, the adaptation of the excitation vectors reflects constraints imposed by the membrane environment since the starting NMs were computed in a homogeneous dielectric medium. Our results showed that the system adaptively adjusted its exploratory directions in response to spatial constraints imposed by the membrane. This observation aligns with prior studies using artificial potentials to mimic membrane effects on protein motion, as demonstrated for glutamate transporters, illustrating that the membrane indeed affects protein motion.^65^

Here, we demonstrate this effect at the atomic level through a method that couples NMA and MD, allowing a comprehensive study of the transition paths between the protein’s macrostates. Numerous methods have been developed to enhance protein sampling. However, techniques like Replica Exchange Molecular Dynamics (REMD) can sometimes lead to nonphysical conformations due to altered energy barriers at modified temperatures.^66^ Similarly, methods utilizing collective variables, such as Targeted Molecular Dynamics (TMD),^67^ may not always follow the lowest energy pathways, potentially traversing energy barriers inaccessible at room temperature. Preserving a protein’s structural integrity is crucial for functional studies, as its native structure has been shown to play an essential role in equilibrium dynamics.^68,69^

Identifying effective CVs is crucial in MD simulations, as optimal CVs can significantly enhance sampling efficiency, whereas suboptimal choices can prolong the time to convergence.^70^ Large-scale functional motions, such as domain shifts, are often characterized by a combination of low-frequency normal modes^37,71^ principal components (PCA)^72^ at the atomistic level or simplified protein models like ENM^73^ or ANM.^74^ Normal modes represent the vibrational spectrum of proteins at a given energetic minimum. Due to the stochastic nature of thermal motions, NM eigenvectors can change as the protein structure evolves into different potential energy wells.^75^ Moreover, displacements along the modes are valid for small distances; however, larger displacements may distort the protein structure if not managed carefully.^61^ To address these challenges and accelerate MD simulations, several methods have been developed that account for low-frequency motions and enhance protein sampling. Among them are ClustENM,^44^ ACM,^33^ ACM-PCA,^60^ NMA-ITS,^62^ which also update the excitation direction during the simulation, as in aMDeNM.

Enhanced sampling methods in MD often employ algorithms that recalculate CVs during simulations. Some methods introduce biases to the potential energy surface to facilitate energy barrier crossing,^62^ while others perform constrained dynamics that directionally guide the sampling.^43,44^ Furthermore, some methods iteratively determine new CVs on the fly using combinations of biased energy potentials.^45^ In contrast, aMDeNM operates without the need of recomputing NMs or introducing biases into the potential terms. Because the potential energy surface is unchanged, the system does not explore regions rendered artificially accessible by a modified potential. Structural distortions and unphysical barrier crossings are substantially reduced. The algorithm evaluates displacements along excitation vectors and updates directions based on the system’s structural and dynamical features. This process naturally activates higher-frequency modes, enabling adaptive sampling that respects energy barriers and avoids excessive distortions or conformations that are unattainable at a given temperature. By aligning excitation directions with intrinsic protein dynamics, aMDeNM enhances sampling efficiency while preserving the system’s secondary structure and internal constraints. The kinetic injection, however, critically unbalances the canonical ensemble by shifting the velocities in a specific direction and therefore does not provide direct equilibrium information. Thus, the method generates excellent structural ensembles for qualitative exploration but cannot straightforwardly yield ΔG, free energy barriers, or transition rates with precision. To recover equilibrium thermodynamics, an additional step is required, as discussed in the original MDeNM paper.^47^ Briefly, free MD simulations can be achieved from relevant conformations obtained by aMDeNM, from which free energy estimates and kinetics can be derived by using population histograms or Markov State Models.^13,47,54,76^

One of the key contributions of our study was demonstrating that low-frequency vibrations (<35 cm^–1^) are closely associated with critical large-scale conformational changes in the protein. Specifically, the FFT analysis of the time series of projected coordinates along aMDeNM trajectories of T4L revealed substantial contributions from low-frequency vibrations, particularly during critical conformational transitions. This finding highlights the importance of stronger couplings at low frequencies for significant structural changes to occur. Our results align with several studies demonstrating that external perturbations at lower frequencies can induce conformational transitions in proteins, whether through low-frequency harmonic,^77^ anharmonic^78^ or electric field excitations.^79^ Furthermore, several studies have shown that combinations of low-frequency normal modes are more effective in describing conformational transitions than a single mode, highlighting the role of multiple frequencies in complex structural changes, such as those involved in allosteric modulations.^80–82^

## 5 Conclusions

In recent years, the field of molecular dynamics has seen the emergence of numerous enhanced sampling methods to improve the exploration of the free-energy landscape.^36,45,83–86^ These approaches range from biasing energy potentials to employing deep-learning algorithms to extract collective variables that can accelerate sampling.

In this context, we introduced new implementations in the MDeNM method for automatically controlling on-the-fly kinetic energy injection, accounting for the natural constraints imposed by the protein structure and its environment during conformational sampling. This adaptive approach prevents structural distortions throughout the simulation, ensuring a more complete and realistic exploration of the conformational space.

Normal modes (NMs) describe the vibrational spectrum of proteins at a given energy minimum. However, because of the stochastic nature of thermal motions, NM eigenvectors can deviate from their original directions as the structure evolves into other potential energy wells. As a result, displacements along the modes are reliable only for small distances; larger displacements, if not handled carefully, may introduce structural distortions. The advantage of our method lies in its ability to adaptively adjust displacement directions, incorporating structural and energetic constraints imposed by the system to facilitate the exploration of new pathways while maintaining the structural integrity.^87^

Our findings indicate that the efficient generation of new excitation directions is achieved by activating different frequencies across the system’s vibrational spectrum. This activation follows the accumulation of energy along the excitation vector and adaptively redirects it toward a more energetically relaxed path. This mechanism is crucial for avoiding the sampling of high-energy barriers that would otherwise lead to distorted structures.

The aMDeNM method effectively accounts for structural constraints in various systems, making it a powerful tool for mapping energy barriers during conformational sampling. Our results highlight the broad applicability of aMDeNM, including its potential for protein–protein flexible docking, structural interpretation of experimental data, atomistic-level conformational exploration of large systems, and identification of transition paths. The aMDeNM code, along with usage instructions, is available at https://github.com/pedro-tulio/amdenm.

## 6 Methods

### 6.1 Probed Systems and Simulation Details

#### 6.1.1 Atomic Coordinates and Experimental Data Sets

The atomic coordinates of bacteriophage T4 lysozyme (PDB ID: 178L),^88^ human calmodulin (PDB ID: 1CLL),^89^ and *Staphylococcus aureus* membrane-bound transglycosylase (PDB ID: 3VMQ).^59^ For structure 3VMQ, a gap between residues I123 and V128 was modeled using MODELER 9.19.^90^ Co-crystallized water molecules were retained in the final models. Hydrogen atoms were added or removed from all structures to alter the protonation state of residues using PROPKA3,^91^ at pH 7.4.

#### 6.1.2 Molecular Dynamics

The simulation systems for T4L and CaM were built using the CHARMM-GUI input generator.^92,93^ MD simulations were performed with CHARMM c41b1 ^94,95^ using the CHARMM36 ^96^ force field with explicit TIP3 water molecules.^97^ Periodic boundary conditions were applied, and electrostatic interactions were treated using the Particle Mesh Ewald (PME) algorithm.^98^ The protein was placed at least 10 Å from the boundaries of an octahedral box, and counterions were added to achieve charge neutrality. We performed 500 steps of steepest descent (SD) followed by 500 steps of Adopted Basis Newton–Raphson (ABNR) energy minimization. Then, NVT equilibration dynamics was carried out using the Velocity Verlet integrator with an integration time of 1 fs during 25 ps. The Nosé-Hoover thermostat was used to maintain a constant temperature.^99,100^ The SHAKE algorithm^101^ was applied to constrain bonds involving hydrogen atoms. Harmonic restraints were applied to protein-heavy atoms to prevent artificial structural distortions, with a force constant of 2.0 kcal/mol^–1^Å^–2^ for backbone atoms and 0.2 kcal/mol^–1^Å^–2^ for side chain heavy atoms. After equilibration, an unconstrained NPT production MD simulation was conducted using the Leapfrog Verlet integrator for 125 ns, maintaining a constant temperature of 303 K and a constant pressure of 1 atm via the Nosé-Hoover algorithm.

We used the CHARMM-GUI membrane builder^102–104^ to generate MD inputs for simulations of the MTG system using GROMACS 5.0.5.^105^ The system was modeled using the CHARMM36^96^ force field. The protein was inserted into a membrane bilayer following OPM orientation.^106^ The membrane composition was set according to data from Hayami et al.^107^ consisting of 43% palmitoyl-oleoyl-phosphatidylglycerol (POPG), 30% palmitoyl-linoleoyl-phosphatidylglycerol (PLPG), 22% 1,1′,2,2′-tetraoleoyl cardiolipin (TOCL1), and 5% of phosphatidic acid (POPA). The system was solvated with explicit TIP3 water molecules and potassium ions in a tetragonal box, ensuring the protein was at least 17.5 Å away from the box boundaries. After 5000 steps of SD energy minimization, the equilibration protocol was adapted from Jo et al.^103^ Finally, an NPT production MD simulation was carried out for 125 ns using the Leapfrog Verlet integrator with all constraints removed. Temperature and pressure were maintained at 303 K and 1 atm, respectively, using the Nosé-Hoover algorithm.

#### 6.1.3 Normal Modes Calculations

The final MD structures of each system were used to carry out energy minimization and NM calculations in vacuum using CHARMM c41b1 ^94,108^ and CHARMM36 ^96^ force fields. van der Waals interactions were calculated up to 10 Å, with an approximation used between 10 and 12 Å via a switching function. Electrostatic interactions were calculated up to 10 Å with a distance-dependent dielectric constant (*ε = 2r*_*ij*_). We used the SD and the Conjugate Gradients (CG) methods to minimize the energy of the structures. This was followed by additional energy minimization using the ABNR algorithm to reach a root-mean-square energy gradient of less than 10^–5^ kcal mol^–1^ Å^–1^.

#### 6.1.4 MDeNM/aMDeNM Simulations

This study compared conformational sampling of three different MDeNM methods: 1) MDeNM in which each kinetic energy input was followed by a relaxation period; 2) cMDeNM in which the kinetic energy input was adjusted to remain constant; and 3) aMDeNM, in which, in addition to maintaining a constant excitation kinetic energy, the orientation of the excitation vector was also adjusted. These methods were applied to the three molecular systems described above and were complemented by and compared with free molecular dynamics simulations. We analyzed to what extent the samplings obtained by these approaches captured the conformational variability of the available experimental structures. In MDeNM, an incremental energy of 5 kcal/mol was added to the system every 2 ps. In contrast, in cMDeNM, a much lower kinetic energy input of 0.125 kcal/mol was applied every 0.1 ps and maintained at this excitation level throughout the simulation, keeping the protein continuously excited and resulting in a significantly reduced relaxation time. It differed from MDeNM in that the relaxation time was much longer. For aMDeNM, we adopted the same values as in cMDeNM, but with the additional feature that the excitation direction was adapted simultaneously with the kinetic energy control (see also the conditions presented in Section 2.2.6).

#### 6.1.5 Well-Tempered Metadynamics

We performed 250 ns of well-tempered metadynamics (WTMetaD)^46^ simulations for T4L and CaM using NAMD3.^109^ We applied the same MD parameters as the previous simulations and used NMs 7 and 8 as collective variables in both systems. We first ran 5 ns of WTMetaD with Colvars active but without biasing the systems. This measures the equilibrium CV distribution and provides the RMSF values needed to set the biasing factor σ for each CV. Thus, we set a biasing factor of σ_T4L_= 6.66 Å and σ_CaM_= 14.20 Å, for T4L and CaM, respectively. We used a Gaussian height of 0.3 kcal/mol, a bias factor of 15, and deposited a Gaussian on the landscape every 1 ps for both systems.

#### 6.1.6 Spectral Analysis

We analyzed the oscillations of the projected coordinate positions along the excitation vectors during the MDeNM, cMDeNM, and aMDeNM simulations to better understand the implications of vibrational movements accompanying the protein’s conformational changes. A spectral analysis was performed for each simulation on a selected excited T4L trajectory. The trajectory was constructed by starting from the initial structure and applying successive, cumulative coordinate displacements along the **Q**_*n*_ vectors, beginning with the lowest frequency mode (NM7). We then calculated the frequency power spectrum as a function of time along the pathway using the Fast Fourier Transform (FFT). Since the FFT requires a trendless curve, we first leveled the curve over time to isolate vibrational motions and eliminate artifacts caused by the continuously increasing values as motion advances along the pathway. The resulting discrete-time trajectory was designated as *d­(t)*. We then constructed spectrograms of *d­(t)*, showing the distribution of power at different frequencies as a function of time. The analysis was limited to a low-frequency band below 35 cm^–1^, avoiding the higher frequencies associated with pressure and heat bath oscillations. Further details on leveling and frequency filtering procedures are provided in the Supporting Information.

To determine how the excitation kinetic energy is absorbed and distributed within a frequency range going up to 35 cm^–1^, we estimated the relative energy contribution of these frequencies using the equation:

11 $$E_{vib}=\frac{1}{N}\overset{N}{\underset{i}{\sum }}A_{i}^{2}$$

where *N* is the number of frequencies up to 35 cm^–1^, and *A*_*i*_ is the corresponding amplitude provided by the FFT for the *i* ^*th*^ frequency.

Formally, an exact vibrational kinetic energy spectrum would require frequency weighting of the spectral amplitudes. However, such weighting strongly amplifies high-frequency noise in the present trajectories. Therefore, the unweighted spectral power was used instead as a robust relative measure of the energy. The MDeNM, cMDeNM, and aMDeNM simulations were started from the same initial excitation vector, and identical time intervals were used for spectral computations to ensure a fair comparison.

#### 6.1.7 Projections of MD Trajectories onto Normal Mode Space

The projection of trajectories into the normal mode space (**q**_proj_) was carried out for each frame following eq 12:

12 $$q_{proj}=M^{1/2}\left(r_{curr}-r_{ref}\right)q_{nm}$$

where ***r***_curr_ is the current position of the system, ***r***_ref_ is the reference position at the start of the excitation cycle, and **q**_nm_ is the normal mode coordinates vector. The current coordinates are superimposed on the reference coordinates before each projection.

## Supplementary Material



## Notes

Additional method details, including aMDeNM variables parametrization description, details on the spectral profile obtention, and protein structural information (PDF)

## ∥

Laboratório de Genética Molecular, Departamento de Medicina Translacional, Faculdade de Ciências Médicas, Universidade Estadual de Campinas (UNICAMP), Rua Tessália Vieira de Camargo 126, 13083–887, Campinas, Brazil; and the Brazilian Institute of Neuroscience and Neurotechnology (BRAINN), Universidade Estadual de Campinas (UNICAMP), Rua Tessália Vieira de Camargo 126, 13083–887, Campinas, Brazil

## ⊥

Laboratory of Computational Biology, National Heart, Lung, and Blood Institute, National Institutes of Health, Bethesda, MD 20892, USA.
