# Supplementary material for: Adaptive Normal Mode Sampling (aMDeNM) Enhances Exploration of Protein Conformational Space and Reveals the Functional Role of Frequency Coupling
Source: J Chem Theory Comput. 2026 Jun 11;22(13):6304–21. doi: 10.1021/acs.jctc.6c00398 (PMC13374046; doi:10.1021/acs.jctc.6c00398)
Supplement: Supplementary file 1 [file ct6c00398_si_001.pdf]

Adaptive Normal Mode Sampling (aMDeNM) Enhances Exploration of Protein Conformational Space and Reveals the Functional Role of Frequency Coupling

Pedro T. Resende-Lara[1,‡,*], *Maurício G. S. Costa*[2], *Balint Dudas*[1,†], *Janka Czigleczki*[3], *Erika Balog*[3], *David Perahia*[1,*]

[1] Laboratoire de Biologie et Pharmacologie Appliquée, Ecole Normale Supérieure Paris-Saclay, 91190 Gif-sur-Yvette, France.

[2] Programa de Computação Científica, Vice-Presidência de Educação, Informação e Comunicação, Fundação Oswaldo Cruz. Av.Brasil 4365, 21040-900, Rio de Janeiro, Brazil.

[3] Department of Biophysics and Radiation Biology, Semmelweis University, 1094 Budapest, Tűzoltó u. 37-47, Hungary.

[‡] Current affiliation: Laboratório de Genética Molecular, Departamento de Medicina Translacional, Faculdade de Ciências Médicas, Universidade Estadual de Campinas (UNICAMP), Rua Tessália Vieira de Camargo 126, 13083-887, Campinas, Brazil; and the Brazilian Institute of Neuroscience and Neurotechnology (BRAINN), Universidade Estadual de Campinas (UNICAMP), Rua Tessália Vieira de Camargo 126, 13083-887, Campinas, Brazil.

[†] Current affiliation: Laboratory of Computational Biology, National Heart, Lung, and Blood Institute, National Institutes of Health, Bethesda, MD 20892, USA.

AUTHOR INFORMATION

Corresponding Author

David PERAHIA

Laboratoire de Biologie et Pharmacologie Appliquée (LBPA)

École Normale Supérieure Paris Saclay

4 Av. des Sciences

91190 Gif-sur-Yvette, France

david.perahia@ens-cachan.fr

Pedro Túlio de RESENDE-LARA

Laboratório de Genética Molecular

Departamento de Medicina Translacional

Faculdade de Ciências Médicas, Universidade Estadual de Campinas (UNICAMP)

Rua Tessália Vieira de Camargo 126, 13083-887, Campinas, Brazil

laraptr@unicamp.br

KEYWORDS

aMDeNM; protein dynamics; conformational sampling; enhanced sampling; collective variables; normal mode analysis; molecular dynamics, spectral analysis, vibrational energy.

SUPPLEMENTARY MATERIAL

1 MONITORING THE KINETIC ENERGY EXCITATION

In this section, we evaluated the performance of cMDeNM and MDeNM on T4 lysozyme (T4L) to assess the impact of kinetic excitation-energy control. In cMDeNM, excitation energy is

maintained at a constant level through frequent, short-time-interval updates. In MDeNM, the excitation energy is applied at longer time intervals, with no control over its level. Each trajectory spanned 50 ps, with excitation energy injection of 0.5 kcal/mol every 0.1 ps along normal mode 7 (NM7), associated with hinge-bending motion. We carried out 3 independent simulations (called here replicas) for both methods, starting from the open conformation of T4L. As noted in the main text, the structure used was PDB 178L, which describes the TL4 open conformation. We observe a rapid, cumulative increase in kinetic energy along the excitation vector in MDeNM, attributed to insufficient relaxation time for the protein to dissipate the added energy completely, leading to structural distortions (black curve, Supplementary Figure 1a). Conversely, cMDeNM maintained low excitation energy levels, effectively preventing such distortions (red curve, Supplementary Figure 1a). Structural analysis, using Cα RMSD relative to the closed conformation (PDB ID: 3FI5[1]), revealed that MDeNM induced a faster transition to the closed state (10 ps) compared to cMDeNM (30 ps) (upper inset in Supplementary Figure 1a). However, the lack of energy control in MDeNM led to overclosure and structural anomalies (black cartoon, Supplementary Figure 1b). In contrast, cMDeNM facilitated a gradual, distortion-free closure, aligning well with experimental data (red cartoon, Supplementary Figure 1c). Additionally, Cα RMSD from the initial structure showed a more rapid increase in MDeNM, while cMDeNM exhibited a steady progression (bottom right panel in Supplementary Figure 1a).

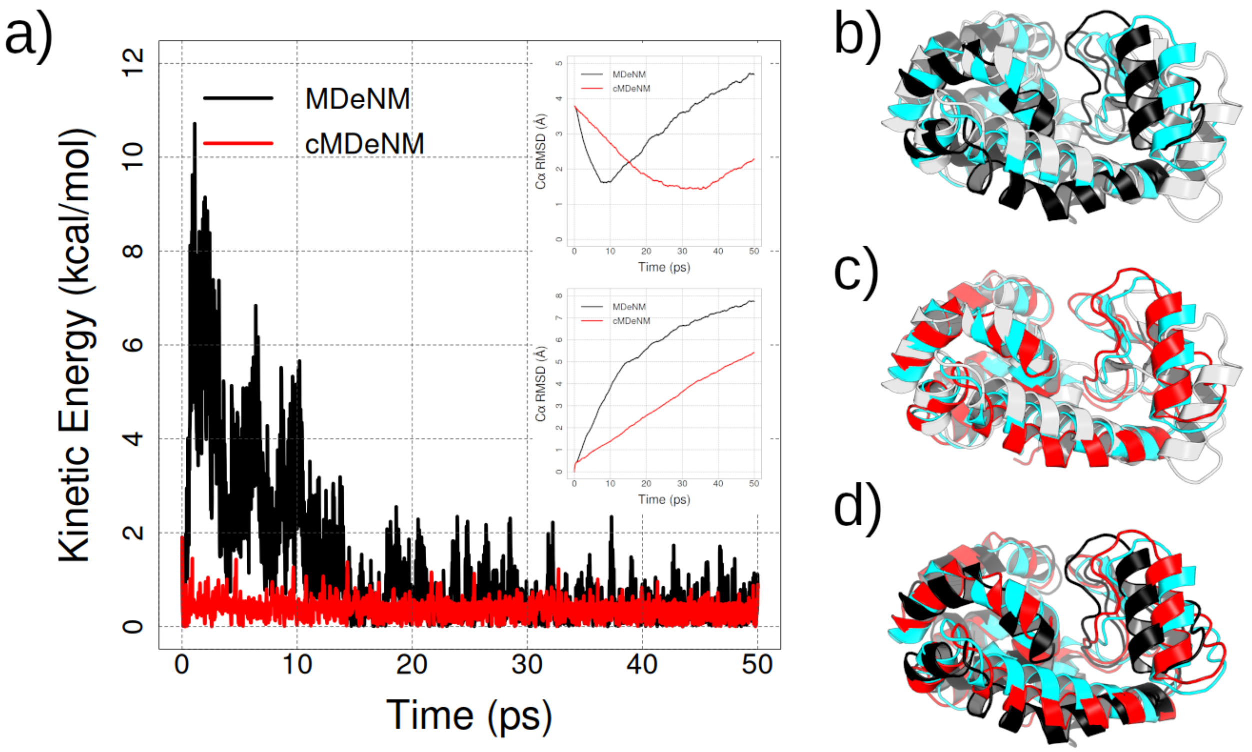

Supplementary Figure 1. Monitoring kinetic energy injection along NM7 in MDeNM and cMDeNM. a) Comparison of kinetic energy along the excitation vector between MDeNM (black) and cMDeNM (red). RMSD of the trajectory's structures from the experimental closed state (upper panel) and the starting position (lower panel). **b)** Initial (light gray) and final (black) conformations of MDeNM run compared with the crystallographic structure 3FI5 (cyan). **c)** Initial (light gray) and final (red) conformations from the cMDeNM simulation, compared with the crystallographic structure 3FI5 (cyan). **d)** Final conformations from MDeNM (black) and cMDeNM (red), both compared with the crystallographic structure 3FI5 (cyan).

Comparisons of the final structures from MDeNM and cMDeNM simulations with the 3FI5 conformation underscore the efficacy of controlled energy injection in achieving accurate structures (Supplementary Figure 1d). These findings highlight the importance of regulating kinetic excitation energy in MDeNM simulations to preserve structural integrity and obtain reliable conformational insights.

2 UPDATE OF EXCITATION DIRECTION

As described in the main text, the correction of the excitation direction in our simulations relies on two key parameters: i) Root-Mean Square Displacement (ℓ): This parameter quantifies the effective displacement of the system along the excitation vector **Q** and determines the frequency at which the algorithm evaluates and potentially adjusts the excitation direction; ii) Deviation Angle ($cos\ \alpha$): this measures the cosine of the angle between the effective displacement vector and the theoretical excitation vector. It serves as a threshold for accepting or rejecting a new excitation direction based on how closely the actual displacement aligns with the theoretical one. To assess the influence of these parameters, we analyzed the hinge-bending motion of T4 lysozyme by computing the Cα RMSD relative to the closed conformation over the simulation trajectories.

We conducted a series of simulations, varying the ℓ values, and terminated each run once the specified ℓ value was reached. This process is detailed in Supplementary Table 1 and illustrated by Supplementary Figure 2. Analysis of the Cα RMSD relative to the closed conformation revealed no significant differences across the ℓ values tested (Supplementary Figure 2a). However, projections of these trajectories onto the excitation vector **Q** yielded $cos\ \alpha$ values ranging from 0.29 to 0.63 (Supplementary Table 1).

Supplementary Table 1. Evaluation of the deviation direction from **Q** vector in different simulation displacements.

ℓ (Å)	0.30	0.35	0.40	0.45	0.50	0.55	0.60	0.65	0.70
cos α	0.29	0.40	0.46	0.48	0.47	0.52	0.57	0.63	0.63

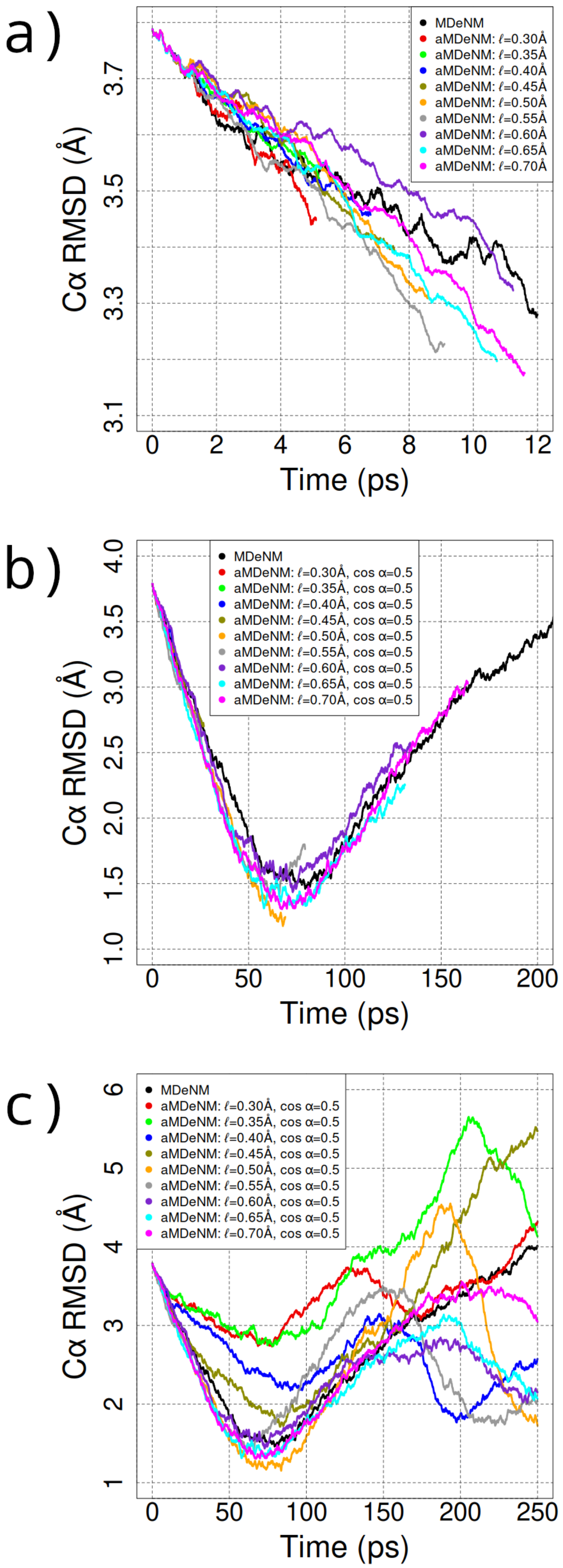
a)
MDeNM
aMDeNM: ℓ=0.30Å
aMDeNM: ℓ=0.35Å
aMDeNM: ℓ=0.40Å
aMDeNM: ℓ=0.45Å
aMDeNM: ℓ=0.50Å
aMDeNM: ℓ=0.55Å
aMDeNM: ℓ=0.60Å
aMDeNM: ℓ=0.65Å
aMDeNM: ℓ=0.70Å
Cα RMSD (Å)
Time (ps)
b)
MDeNM
aMDeNM: ℓ=0.30Å, cos α=0.5
aMDeNM: ℓ=0.35Å, cos α=0.5
aMDeNM: ℓ=0.40Å, cos α=0.5
aMDeNM: ℓ=0.45Å, cos α=0.5
aMDeNM: ℓ=0.50Å, cos α=0.5
aMDeNM: ℓ=0.55Å, cos α=0.5
aMDeNM: ℓ=0.60Å, cos α=0.5
aMDeNM: ℓ=0.65Å, cos α=0.5
aMDeNM: ℓ=0.70Å, cos α=0.5
Cα RMSD (Å)
Time (ps)
c)
MDeNM
aMDeNM: ℓ=0.30Å, cos α=0.5
aMDeNM: ℓ=0.35Å, cos α=0.5
aMDeNM: ℓ=0.40Å, cos α=0.5
aMDeNM: ℓ=0.45Å, cos α=0.5
aMDeNM: ℓ=0.50Å, cos α=0.5
aMDeNM: ℓ=0.55Å, cos α=0.5
aMDeNM: ℓ=0.60Å, cos α=0.5
aMDeNM: ℓ=0.65Å, cos α=0.5
aMDeNM: ℓ=0.70Å, cos α=0.5
Cα RMSD (Å)
Time (ps)

Supplementary Figure 2. Screening of projected coordinate displacement parameter (ℓ) in aMDeNM direction correction by measuring the Cα RMSD from PDB 3FI5. a) Simulations were halted upon reaching the ℓ threshold, revealing no significant RMSD differences. **b)** With *cos α* fixed at 0.50 and varying ℓ**,** the RMSD curve was monitored until the first update in the excitation direction. Smaller ℓ values (red and green curves) led to premature direction updates, while larger ℓ values (cyan and magenta curves) delayed updates excessively. **c)** Depicts the RMSD curve over the entire 250 ps aMDeNM simulation. Smaller ℓ values (red and green curves) led to earlier deviations from the closing motion, whereas higher thresholds resulted in insufficient trajectory correction (cyan and, mainly, magenta curves), leaving excessive closing motion. An ℓ ***value of*** 0.50 (orange curve) achieved the greatest RMSD variation between open and closed conformations without structural deformations. The increase in RMSD observed after the closing motion suggests the effective identification of the relaxation pathway. Data represent averages from three independent replicas of each simulation.

Linear regression analysis of the relationship between ℓ and cosα yields a near-unit slope (p-value < 0.0001), suggesting a linear correlation between the two parameters. This indicates that an optimized combination of both is essential for effectively adjusting the excitation direction to reach the target conformation. Consequently, we set *cos α* to *0.50*, approximately the average of the sampled values, and tested several values of ℓ. Monitoring the hinge-bending trajectory until the first update (i.e., when both ℓ and *cos α* thresholds are met) demonstrated that smaller ℓ values prompt earlier changes in the excitation direction than larger ones (*Supplementary Figure 2b*). Notably, with small ℓ values, the system alters its path before reaching the minimum RMSD value (closest to the 3FI5 configuration). In contrast, larger ℓ values exhibit behavior similar to MDeNM (black curve). This suggests that ℓ should be balanced; values that are too small lead to premature direction changes, while values that are too large hinder proper updates.

In most simulations, the structure reaches either a macrostate or a transition state (Supplementary Figure 2c). For small ℓ values such as 0.30 Å and 0.35 Å (red and green, respectively), the closed state was not achieved. Conversely, for 0.55 Å and 0.60 Å (gray and purple, respectively), it mainly sampled the closed conformation with some drifting between the macrostates. We observed almost no directional change for the highest ℓ values (0.65 Å, cyan, and 0.70 Å, magenta), which occurred near 200 ps of simulation. Between ℓ=0.45 Å and ℓ=0.50 Å (olive and orange, respectively), the latter yielded optimal results. T4L progressed further into the closing motion in these instances, exploring diverse directions while preserving the secondary structure, and could then relax and sample conformations near the closed structure again through a different path. Therefore, we selected $\ell = 0.50$ Å and $\cos \alpha = 0.50$ as the optimal parameters for subsequent simulations.

3 DEFINITION OF RELEVANT INTERNAL COORDINATES FOR THE STUDY CASES

3.1 *T4 lysozyme*

Lysozyme T4L degrades host peptidoglycans, leading to the rupture of the host cell wall and the consequent release of mature viral particles.[2] For decades, it has served as a model for studying proteins' structural and stability factors.[3] T4L comprises 164 residues organized into two domains (N and C), connected by a long α-helix. Its active-site cleft (E11 and D20) lies at the interface between the two domains.[4] Most experimentally resolved T4L conformations adopt a closed form, similar to the peptidoglycan-bound conformation. However, a more open conformation is believed to be necessary for ligand binding. Indeed, Goto *et al.*[5] have observed structures in which the catalytic cleft is approximately 17° more open than in the crystallographic structure 3LZM. Zhang *et al.*[6] have shown that various crystallographic structures exhibit hinge-bending angles exceeding 50 °.

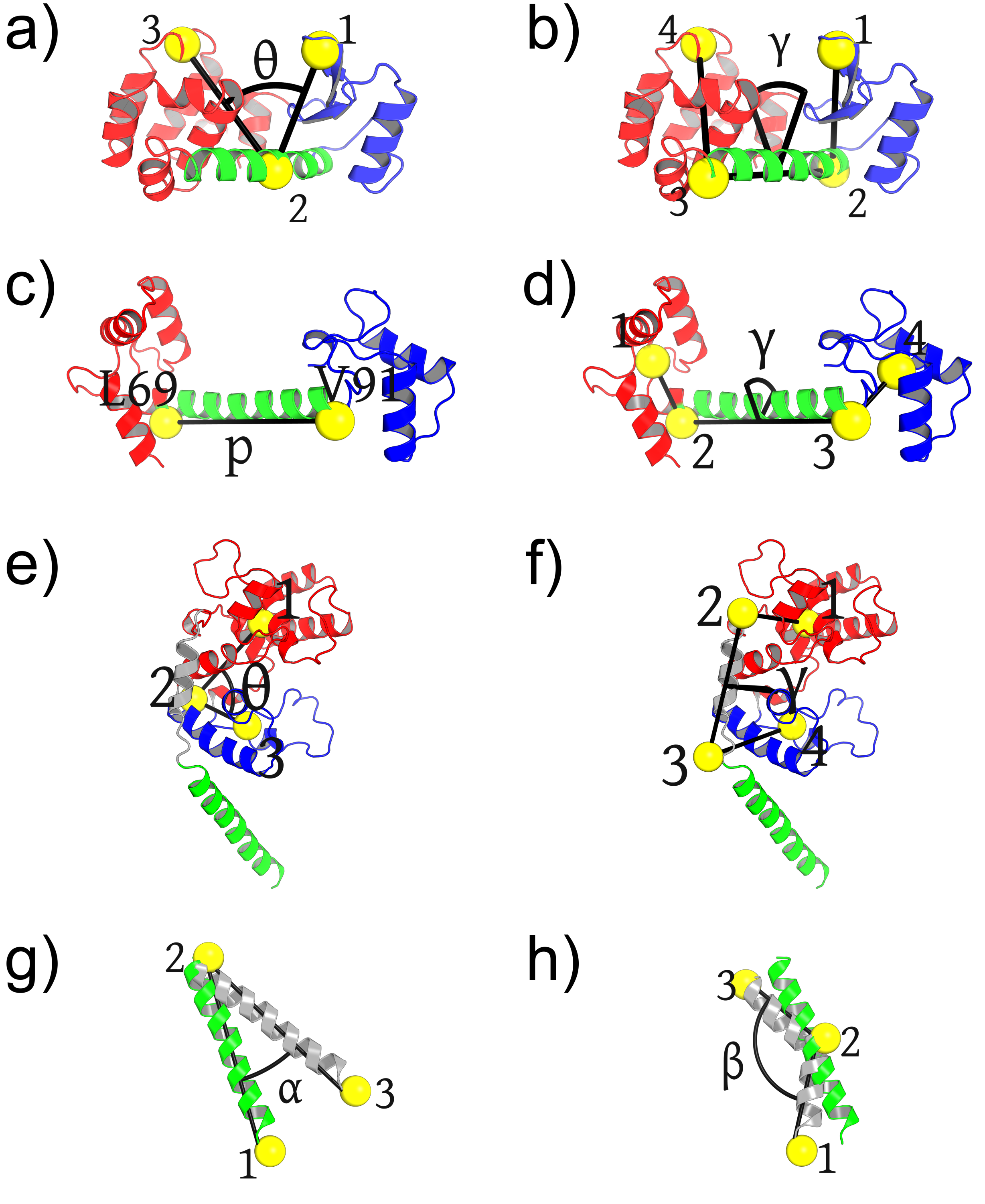

Supplementary Figure 3. Key structural descriptors in the case studies. a) T4L hinge-bending angle, *θ*. **b)** T4L torsion angle, *γ*. **c)** CaM link end-to-end distance, *p*. **d)** CaM torsion angle, *γ*. **e)** MTG globular hinge-bending angle, *θ*. **f)** MTG globular torsion angle, *γ*. **g)** MTG transmembrane helix rotation, *α*. **h)** MTG transmembrane helix bending angle, *β*.

To analyze the dynamics of T4L, we evaluated its hinge bending and torsion motions, as these are the most critical movements for its function.[4,7] The T4L hinge angle θ (Supplementary Figure 3a) was defined as the angle between pseudo-bonds connecting the centers of mass of Cα atoms from three successive regions: (1) residues K19-G23 in the N-domain (blue); (2) residues N68-D72 in the middle of the inter-domain α-helix (green), and (3) residues R137-Q141 in the C-domain (red). The torsion angle γ between T4L domains (Supplementary Figure 3b) was defined as the dihedral angle formed by pseudo-bonds linking the centers of mass of Cα atoms of: (1) residues K19-G23 in the N-domain (blue); (2) residue D61 at the beginning of the inter-domain α-helix; (3) residue L79 at the end of the inter-domain α-helix and (4) residues R137-Q141 in the C-domain.

3.2 *Calmodulin*

Calmodulin (CaM) is a Ca^{2+}–binding protein that regulates the activity of numerous partners, including protein kinases, NAD kinases, phosphodiesterases, calcium pumps, and G proteins, among others.[8] CaM consists of two domains, each formed by EF-hand helix-loop-helix motifs, connected by a flexible inter-domain linker. In the calcium-free state (apo-CaM), the two domains remain collapsed together, adopting a closed conformation in which the linker is partially or fully disordered.[9,10] When Ca^{2+} ions bind (Ca^{2+}–CaM), the domains separate, exposing a hydrophobic cleft that significantly enhances binding affinity for various partners.[9] Although the inter-domain linker can adopt an α-helical structure, it remains highly flexible, allowing a wide range of inter-domain orientations. This flexibility is influenced by various factors, particularly the modulation of electrostatic interactions within the highly flexible inter-domain linker between the two CaM domains.[11] These electrostatic adjustments determine whether the linker adopts a compact or extended conformation, ultimately regulating CaM's ability to bind partner proteins.[10,12]

As described by Aykut *et al.*,[11] the domain torsion and linker end-to-end distance are two

effective measures for identifying different conformational states of CaM. The end-to-end linker distance p is computed as the distance between the Cα atoms of residues L69 and V91, as shown in Supplementary Figure 3c. The torsion angle γ between CaM domains is defined by the dihedral angle formed between the centers of mass of Cα atoms from the following regions: (1) residues Q8-F68 in the N-domain; (2) residue L69 at the beginning of the inter-domain linker; (3) residue V91 at the end of the inter-domain linker; and (4) F92-V142 in the C-domain. This is illustrated in Supplementary Figure 3d.

3.3 *Monofunctional transglycosylase*

MTG is a membrane-bound peptidoglycan polymerase that catalyzes glycan chain elongation, which is essential to synthesizing bacterial cell walls.[13] The transmembrane (TM) helix plays a crucial role in the MTG's activity by facilitating hydrophobic interactions and ensuring the enzyme's proper orientation within the membrane for effective catalysis.[14] This positioning brings the jaw and head subdomains of MTG closer to each other, enabling the formation of the lipid-binding pocket.[13] Although few MTG structures have been resolved, the available apo and ligand-bound structures reveal a consistent orientation within the lipid bilayer, aligning well with its *E. coli* analog, penicillin-binding protein 1b.[14] Given its essential role in bacterial survival, MTG has become a promising target for developing antibiotics. However, despite recent advances in understanding its transglycosylation mechanism, developing new structure-based antibiotics targeting this enzyme remains an ongoing challenge.

We considered the hinge angle θ, which is defined by the pseudo-bonds linking the centers of mass of Cα atoms from the following regions: (1) residues R67-G130 and N168-R269 in the head domain; (2) N106 and Y167 interconnecting the two globular domains; and (3) H107-R126 in the jaw domain. These regions are shown in Supplementary Figure 3e. We also computed the torsion angle γ (Supplementary Figure 3f) defined by the dihedral angle formed between the centers of mass of Cα atoms from the MTG regions: (1) residues R67-G130 and N168-R269 in

the head domain; (2) residue R67; (3) residue S80; and (4) H107-R126 in the jaw domain. Given the critical role of the TM helix in MTG's enzymatic activity, we also considered its orientation and bending within the membrane. The orientational variations were quantified by tracking changes in the angle α, representing the angle the TM helix's longitudinal axis makes relative to itself over time, as depicted in Supplementary Figure 3g. At the same time, the bending of the TM helix was estimated by the angle β defined by the Cα atoms of residues R41, I53, and R67 as shown in Supplementary Figure 3h. In addition, we independently analyzed the conformational sampling of the globular domains.

4 SECONDARY STRUCTURE

In addition to the main text, we evaluated the secondary structure conservation along the MDeNM, cMDeNM, and aMDeNM trajectories. The concern about structural distortions arises when an extensive sampling method, such as aMDeNM, is used. Considering the α-helix content of the experimental dataset of T4L (Supplementary Figure 4a), we observed that this structural motif was better preserved in the free MD and MDeNM (Supplementary Figure 4b and 4c, respectively). In contrast, the cMDeNM presented a relative decrease due to deformations caused by the excessive closing of the protein (Supplementary Figure 4d). The adaptive approach presented some structures with intermediate values (Supplementary Figure 4e). Still, the general features of the experimental structures were also present. Experimental data show that the majority of structures contain approximately 12 residues in the β-sheet (Supplementary Figure 4f). While the free MD shows some loss of residues in the β-sheet (Supplementary Figure 4g), MDeNM, cMDeNM, and aMDeNM present a similar content proportion of this motif (Supplementary Figure 4h, 4i, and 4j, respectively). All MDeNM approaches also yield conformations with more than 15 residues in β-sheet, indicating a preference for structuration during the exploration of large-scale motions.

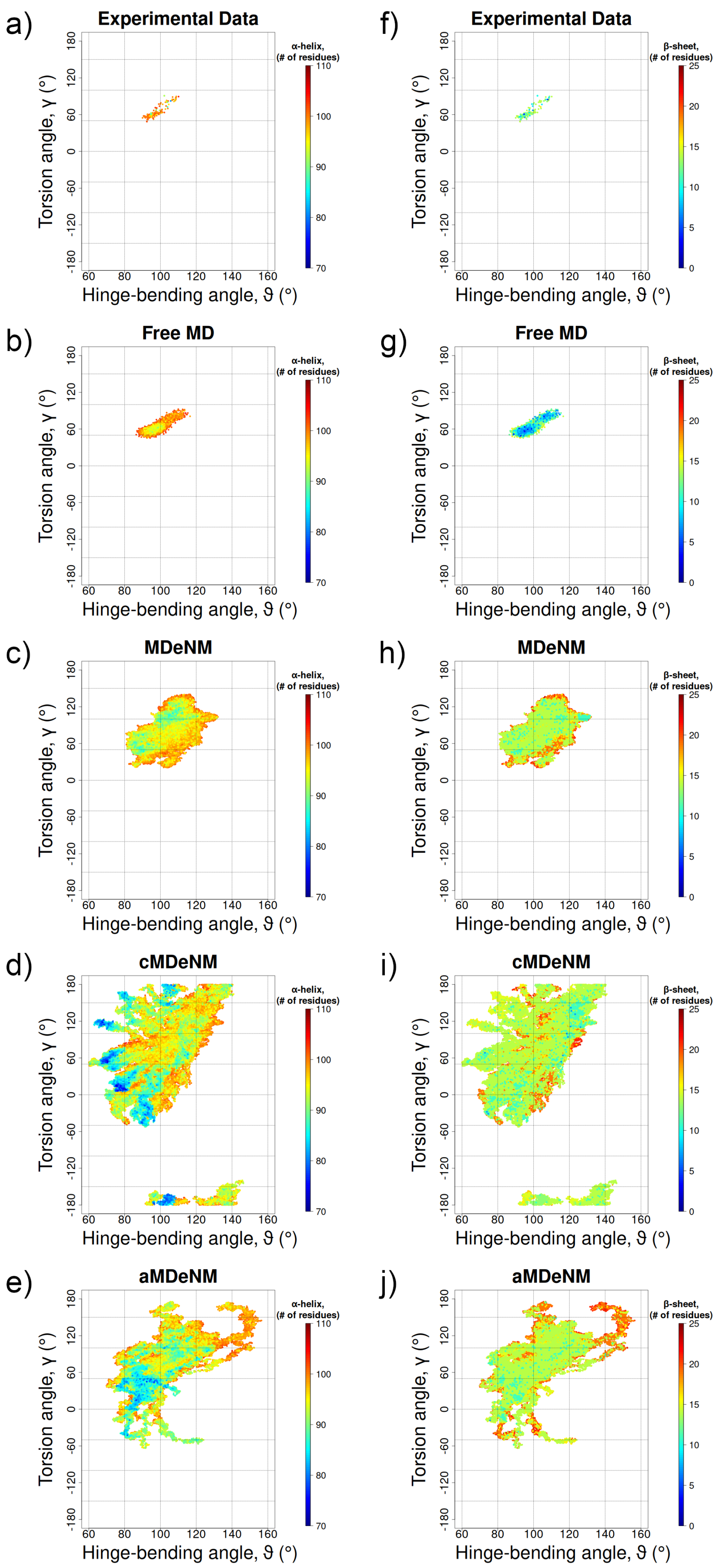
a)
Experimental Data
b)
Free MD
c)
MDeNM
d)
cMDeNM
e)
aMDeNM
f)
Experimental Data
g)
Free MD
h)
MDeNM
i)
cMDeNM
j)
aMDeNM
Torsion angle, γ (°)
Hinge-bending angle, ϑ (°)
α-helix, (# of residues)
β-sheet, (# of residues)
-180
-120
-60
0
60
120
180
60
80
100
120
140
160
70
80
90
100
110
0
5
10
15
20
25

Supplementary Figure 4. DSSP secondary structure assessment for T4L. Heatmaps of α-helix content for **a)** experimental data, **b)** free MD, **c)** MDeNM, **d)** cMDeNM, and **e)** aMDeNM. While cMDeNM presented a significant loss of helix content, MDeNM and aMDeNM maintained greater stability. Heatmaps of β-Sheet content for **f)** experimental data, **g)** free MD, **h)** MDeNM, **i)** cMDeNM, and **j)** aMDeNM. All MDeNM approaches showed similar results for β-sheet content.

CaM structures exhibit a highly heterogeneous α-helix content (Supplementary Figure 5a), primarily due to the flexibility of its inter-domain linker, which can adopt helical or disordered structures. This flexibility is crucial for CaM's ability to sample a large ensemble of conformations, facilitating effective signal transmission and calcium binding. As expected, free MD, MDeNM, and cMDeNM (Supplementary Figure 5b, 4c and 4d, respectively) oversample well-structured conformations, failing to capture states with reduced helical content. In contrast, aMDeNM approach, which updates the excitation vector during simulations, enables the protein to explore different pathways and, therefore, alter its secondary structure (Supplementary Figure 5e). This results in a broader sampling that aligns more closely with the RMSD values presented in the main text and with experimental observations. Regarding β-sheet content, results are very similar to those presented for the α-helix; i.e., the experimental dataset shows a heterogeneous pattern (Supplementary Figure 5f) that is not fully reproduced by free MD, MDeNM, and cMDeNM (Supplementary Figure 5g, 4h, and 4i, respectively). Again, aMDeNM simulations closely mirror experimental results, capturing the variability in β-sheet content (Supplementary Figure 5j).

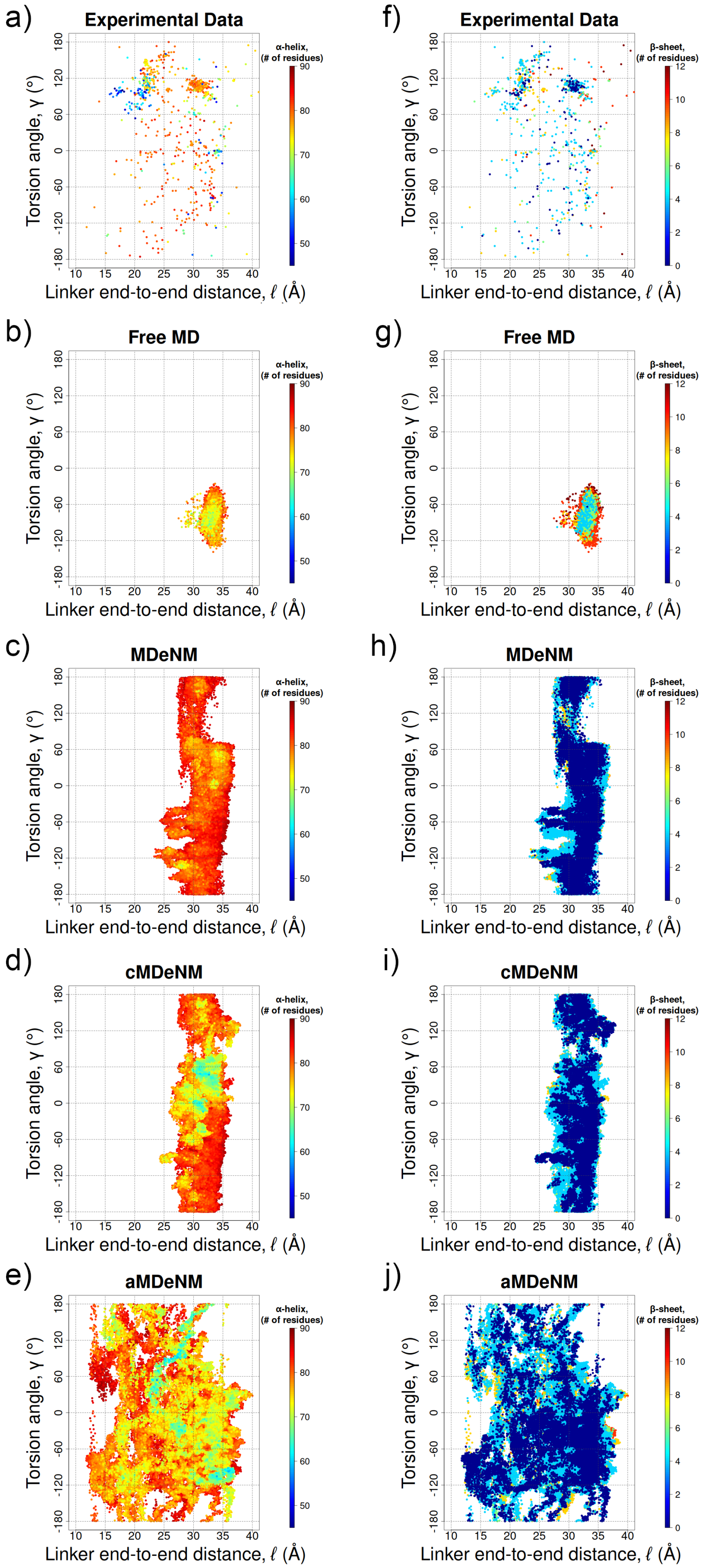
a)
Experimental Data
α-helix, (# of residues)
f)
Experimental Data
β-sheet, (# of residues)
b)
Free MD
g)
Free MD
c)
MDeNM
h)
MDeNM
d)
cMDeNM
i)
cMDeNM
e)
aMDeNM
j)
aMDeNM
Torsion angle, γ (°)
Linker end-to-end distance, ℓ (Å)
180
120
60
0
-60
-120
-180
10
15
20
25
30
35
40
90
80
70
60
50
12
10
8
6
4
2
0

Supplementary Figure 5. DSSP secondary structure assessment for CaM. Heatmaps of α-helix content for **a)** experimental data, **b)** free MD, **c)** MDeNM, **d)** cMDeNM, and **e)** aMDeNM. β-Sheet content for **f)** experimental data, **g)** free MD, **h)** MDeNM, **i)** cMDeNM, and **j)** aMDeNM. aMDeNM demonstrated strong agreement with the experimental dataset, whereas the other approaches failed to accurately reproduce the experimental profile.

5 PROJECTED COORDINATES TRAJECTORY LEVELLING AND NOISE FILTERING

The projected coordinates trajectory was subdivided into different short segments, *D(n,m)*, each of them corresponding to an update of the excitation vector; *n* refers to the excitation vector numbering from the beginning of the simulation to the end (i.e., *n=1* the first excitation direction which corresponds to a given combination of NM vectors, *n=2* the second updated one, etc.); *m* refers to the relative numbering of the excitation kinetic energy update, to keep it constant, within each vector updating stage. *M* is the total number of kinetic updates within each stage, *m* running from 1 to *M*.

To process the RMSD data, we employed the following methodology:

5.1 Linear approximation of D(n,m) curve

We applied a linear approximation to the curve to level it up, following the equation:

$$d(n,m,t) = D(n,m) - D(n-1,M) - m\left(\frac{D(n,M) - D(n-1,M)}{M}\right)$$

5.2 Construction of a discretized time series

The discretized time series *s(t)* comprises successive values of *S(n,m)*, over the ranges of *n = 1 to 20* and *m = 1 to 125*, with a fixed time interval of 0.1 ps between points. The unleveled and leveled curves are presented in Supplementary Figure 6a as the black and red curves, respectively.

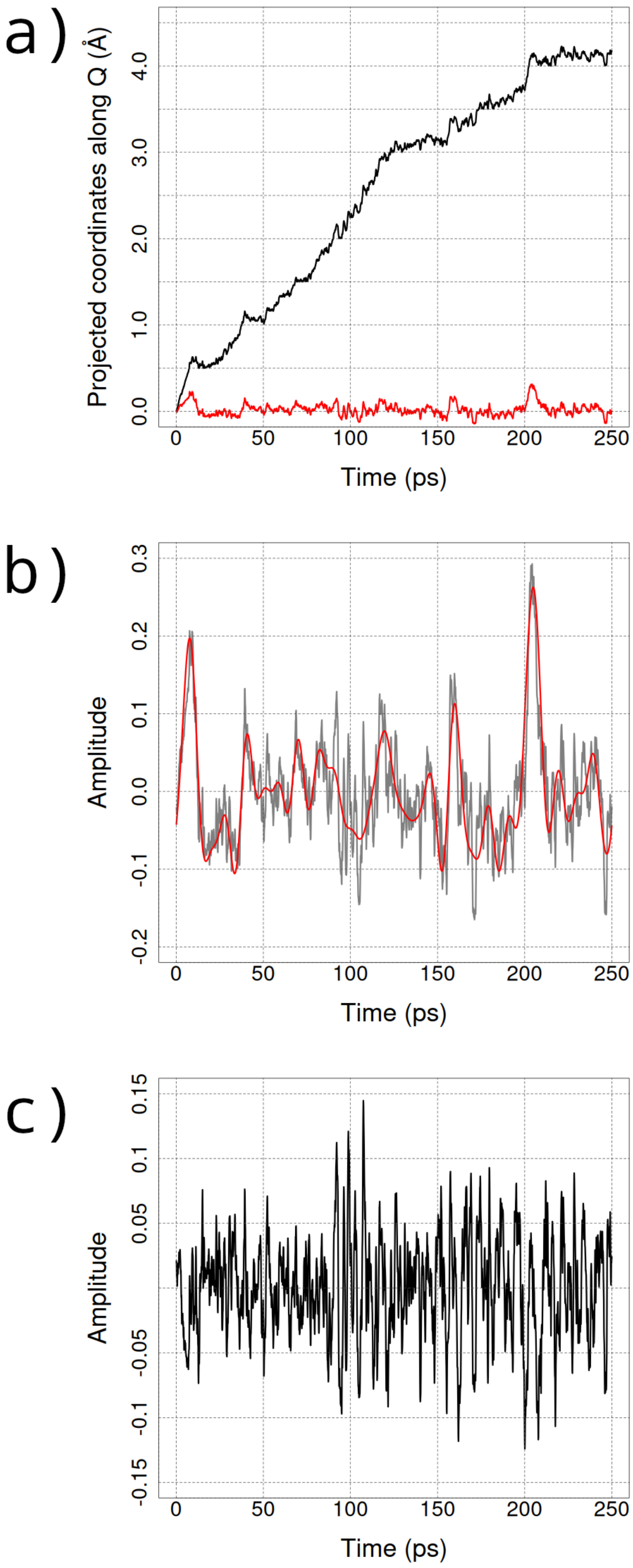

a)
b)
c)
Projected coordinates along Q (Å)
Amplitude
Time (ps)

Supplementary Figure 6. Spectrogram signal processing. The projected coordinates on the excitation vectors are leveled and filtered. **a)** Discretized time series of the coordinate displacements (black curve) and its leveled trajectory (red curve). **b)** Time series of the coordinate displacements from the average (black curve) and the signal after 0.01 Hz low-pass filtering (red curve). **c)** Processed signal: the difference between the average time-series and the filtered signal.

5.3 Deviation from the average

The time series of the variations from the average, $d(t) = s(t) - \langle s(t) \rangle$, is established for further analysis as shown in Supplementary Figure 6b (gray curve).

5.4 Noise reduction via low-pass filtering

To attenuate noise due to impulses initiated by the excitation kinetic energies and directional changes that correspond to very low frequencies, a 0.01 Hz low-pass filter was applied to *d(t)* using the package phonTools[15] available on R 3.4.4[16], producing the red curve in Supplementary Figure 5b.

5.5 Signal refinement

The filtered signal was subtracted of *d(t)* to obtain the final processed signal as shown in Supplementary Figure 6c.

5.6 Spectral analysis

We performed a Fast Fourier Transform (FFT) on the processed signal and generated a spectrogram using the signal[17] package in R. Plots were created using the oce[18] package in R, as detailed in the main text.

6 SUPPLEMENTARY REFERENCES

(1) Mooers, B. H. M.; Baase, W. A.; Wray, J. W.; Matthews, B. W. Contributions of All 20 Amino Acids at Site 96 to the Stability and Structure of T4 Lysozyme. *Protein Sci.* **2009**, *18* (5), 871–880. https://doi.org/10.1002/pro.94.

(2) Moussa, S. H.; Kuznetsov, V.; Tran, T. A. T.; Sacchettini, J. C.; Young, R. Protein

Determinants of Phage T4 Lysis Inhibition. *Protein Sci.* **2012**, *21* (4), 571–582. https://doi.org/10.1002/pro.2042.

(3) Baase, W. A.; Liu, L.; Tronrud, D. E.; Matthews, B. W. Lessons from the Lysozyme of Phage T4. *Protein Sci.* **2010**, *19* (4), 631–641. https://doi.org/10.1002/pro.344.

(4) Yirdaw, R. B. B.; McHaourab, H. S. S. Direct Observation of T4 Lysozyme Hinge-Bending Motion by Fluorescence Correlation Spectroscopy. *Biophys. J.* **2012**, *103* (7), 1525–1536. https://doi.org/10.1016/j.bpj.2012.07.053.

(5) Goto, N. K.; Skrynnikov, N. R.; Dahlquist, F. W.; Kay, L. E. What Is the Average Conformation of Bacteriophage T4 Lysozyme in Solution? A Domain Orientation Study Using Dipolar Couplings Measured by Solution NMR. *J. Mol. Biol.* **2001**, *308* (4), 745–764. https://doi.org/10.1006/jmbi.2001.4614.

(6) Zhang, X.; Wozniak, J. A.; Matthews, B. W. Protein Flexibility and Adaptability Seen in 25 Crystal Forms of T4 Lysozyme. *J. Mol. Biol.* **1995**, *250* (4), 527–552. https://doi.org/10.1006/JMBI.1995.0396.

(7) De Groot, B. L.; Hayward, S.; Van Aalten, D. M. F.; Amadei, A.; Berendsen, H. J. C. Domain Motions in Bacteriophage T4 Lysozyme: A Comparison between Molecular Dynamics and Crystallographic Data. *Proteins Struct. Funct. Genet.* **1998**, *31* (2), 116–127. https://doi.org/10.1002/(SICI)1097-0134(19980501)31:2<116::AID-PROT2>3.0.CO;2-K.

(8) Ikura, M.; Ames, J. B. Genetic Polymorphism and Protein Conformational Plasticity in the Calmodulin Superfamily: Two Ways to Promote Multifunctionality. *Proc. Natl. Acad. Sci.* **2006**, *103* (5), 1159–1164. https://doi.org/10.1073/PNAS.0508640103.

(9) Gsponer, J.; Christodoulou, J.; Cavalli, A.; Bui, J. M.; Richter, B.; Dobson, C. M.; Vendruscolo, M. A Coupled Equilibrium Shift Mechanism in Calmodulin-Mediated Signal Transduction. *Structure* **2008**, *16* (5), 736–746. https://doi.org/10.1016/j.str.2008.02.017.

(10) Pandey, K.; Dhoke, R. R.; Rathore, Y. S.; Nath, S. K.; Verma, N.; Bawa, S.; Ashish. Low PH Overrides the Need of Calcium Ions for the Shape-Function Relationship of Calmodulin: Resolving Prevailing Debates. *J. Phys. Chem. B* **2014**, *118* (19), 5059–5074. https://doi.org/10.1021/jp501641r.

(11) Aykut, A. O.; Atilgan, A. R.; Atilgan, C. Designing Molecular Dynamics Simulations to Shift Populations of the Conformational States of Calmodulin. *PLOS Comput. Biol.* **2013**, *9* (12), e1003366. https://doi.org/10.1371/JOURNAL.PCBI.1003366.

(12) Kukic, P.; Camilloni, C.; Cavalli, A.; Vendruscolo, M. Determination of the Individual Roles of the Linker Residues in the Interdomain Motions of Calmodulin Using NMR Chemical Shifts. *J. Mol. Biol.* **2014**, *426* (8), 1826–1838. https://doi.org/10.1016/j.jmb.2014.02.002.

(13) Heaslet, H.; Shaw, B.; Mistry, A.; Miller, A. A. Characterization of the Active Site of S. Aureus Monofunctional Glycosyltransferase (Mtg) by Site-Directed Mutation and Structural Analysis of the Protein Complexed with Moenomycin. *J. Struct. Biol.* **2009**, *167* (2), 129–135. https://doi.org/10.1016/j.jsb.2009.04.010.

(14) Huang, C.-Y.; Shih, H.-W.; Lin, L.-Y.; Tien, Y.-W.; Cheng, T.-J. R.; Cheng, W.-C.; Wong, C.-H.; Ma, C. Crystal Structure of Staphylococcus Aureus Transglycosylase in Complex with a Lipid II Analog and Elucidation of Peptidoglycan Synthesis Mechanism. *Proc.*

Natl. Acad. Sci. U. S. A. **2012**, *109* (17), 6496–6501. https://doi.org/10.1073/pnas.1203900109.

(15) Barreda, S. PhonTools: Functions for Phonetics in R. 2015.

(16) R Core Team. R: A Language and Environment for Statistical Computing. *R Foundation for Statistical Computing*. Vienna 2016. http://www.r-project.org/.

(17) signal developers. Signal: Signal Processing. 2014. http://r-forge.r-project.org/projects/signal/.

(18) Kelley, D.; Richards, C.; Layton, C.; Survey, B. G. Oce: Analysis of Oceanographic Data. 2020.
